# Global epidemiological and aetiological patterns of hand, foot, and mouth disease: a country-level scoping synthesis

**DOI:** 10.7189/jogh.16.04220

**Published:** 2026-07-17

**Authors:** Zhen Li, Xue Han, Yuan Ren

**Affiliations:** 1 Strategic Marketing Centre, Sinovac Biotech Co., Ltd., Beijing, China; 2 Strategic Marketing Centre, Sinovac Holding Group Co., Ltd., Beijing, China

**Keywords:** hand, foot, and mouth disease, HFMD, scoping synthesis, epidemiology, aetiology, country-level

## Abstract

**Background:**

Hand, foot, and mouth disease (HFMD) has historically been predominantly reported in the Asia-Pacific region, while its global epidemiology and aetiology remain fragmented and incompletely characterised. We aimed to synthesise currently available country-level evidence to describe global epidemiological and aetiological patterns of HFMD.

**Methods:**

We conducted a country-level scoping synthesis of published studies and publicly available surveillance reports through August 2025. Studies were eligible for inclusion if they reported epidemiological or aetiological data on HFMD at the national or subnational level, including case numbers, incidence, outbreaks, and enterovirus (EV) serotype distributions. We qualitatively synthesised the data, with a focus on surveillance availability, epidemiological trends, and major circulating EV serotypes.

**Results:**

Reported HFMD incidence declined during the COVID-19 pandemic and subsequently rebounded in many settings, in some settings exceeding pre-pandemic levels. Aetiological evidence suggests an increasing circulation of coxsackievirus A6 in multiple regions, with coxsackievirus A16 and EV A71 continuing to co-circulate in specific settings. However, routine epidemiological and aetiological surveillance remained largely concentrated in the Asia-Pacific region. In many other countries, available data were limited and primarily derived from outbreak-driven or sporadic studies. Heterogeneity was observed in surveillance systems and data availability across countries.

**Conclusions:**

Global HFMD epidemiological and aetiological patterns are characterised by disparities in surveillance capacity and data availability. Developing standardised surveillance and aetiological monitoring is essential to further understand the global landscape of HFMD, improve data comparability, support early detection of epidemiological changes, and inform evidence-based prevention and control strategies.

Hand, foot, and mouth disease (HFMD) was first described in 1948 [1], and the earliest documented outbreak occurred in 1957 in Toronto, Canada [2]. Historically, this disease received limited attention due to its typically self-limiting clinical course [1]. However, over the past two decades, accumulating evidence has highlighted HFMD as a significant public health concern, particularly due to outbreaks associated with severe complications [1,3].

Since 1997, large-scale outbreaks of enterovirus (EV)-associated HFMD have been reported across several countries in the Asia-Pacific region, including Malaysia, Japan, China, Vietnam, and Singapore [1]. Following 2010, a notable annual increase in HFMD-related publications has been observed, with the majority originating from this region [4]. Nevertheless, HFMD outbreaks and case reports have also been documented in other regions, including North America and Europe [1,3,5], suggesting a broader geographical distribution than previously recognised.

The aetiology of HFMD is complex and involves multiple EV serotypes, with EV-A71, coxsackievirus (CV)-A16, CV-A6, and CV-A10 being the most commonly reported and responsible for outbreaks and epidemics [6,7]. Other serotypes, such as CV-A2, CV-A4, CV-A5 and CV-B1 to CV-B5, have also been reported, although less frequently [7,8].

In earlier years, EV-A71 and CV-A16 were considered the predominant serotypes causing HFMD, typically exhibiting a two- to three-year cyclical outbreak pattern, particularly in the Asia-Pacific region [1,9]. In recent years, however, several studies have reported a shift in the major circulating serotypes, with increasing detection of CV-A6 and CV-A10 across multiple settings [1,6,9]. Additionally, other serotypes, including CV-A4, CV-B3 and CV-B5, have been increasingly reported [1,6], although their epidemiological significance remains uncertain.

Despite numerous studies on HFMD [4], the global understanding of its epidemiology and circulating serotypes remains highly fragmented. Existing reviews [1,8,9] primarily focused on the general epidemiology and aetiology, clinical features, complications, as well as health and economic burdens associated with HFMD, especially in East and Southeast Asia. Comprehensive syntheses that systematically describe epidemiological trends and aetiological patterns at a country level remain limited.

In early 2025, outbreaks were reported in several countries and territories outside the Asia-Pacific region, including Guyana, Mexico, Peru, Trinidad and Tobago, and the USA Virgin Islands [10], alongside continued HFMD outbreaks reported in Asian countries, such as Malaysia [11–13], Thailand [14,15], and the Philippines [16–18]. These reports highlighted the continued occurrence of HFMD, and it is essential to remain vigilant about the potential risk, as emphasised by the Pan American Health Organisation and the World Health Organisation (WHO) [10].

A comprehensive understanding of the current epidemiology and aetiology of HFMD is therefore necessary to identify data gaps and research priorities, as well as to guide prevention and control strategies. Hence, we aimed to provide a country-level synthesis of global HFMD epidemiological and aetiological patterns.

## METHODS

In conducting the study, we adhered to JoGH’s GRABDROP guidelines [19] (Table S1 in **Online Supplementary Document**). We designed the study as a country-level scoping synthesis to summarise publicly available epidemiological and aetiological data on HFMD. Data sources included published literature and publicly accessible surveillance data from official websites, such as the Centres for Disease Control and Prevention and national Ministries of Health.

### Study selection and eligibility criteria

We conducted a literature search using the Foreign Medical Literature Retrieval Service (FMRS) (Shenzhen METSTR Technology Co., Ltd, Shenzhen, China), an integrated literature retrieval platform that indexes multiple international bibliographic databases, including PubMed and Embase, and covers more than 35,000 international journals. We designed the search strategy to achieve broad coverage comparable to direct searches of those primary databases. Since FMRS is not a widely recognised standard database, we validated the search results by conducting parallel searches in PubMed using the same search terms and time frame to assess their reliability and completeness. The findings showed a high degree of consistency between the two approaches, suggesting broadly comparable coverage to that of direct database searches.

We considered studies as eligible for inclusion if they reported epidemiological or aetiological data on HFMD at the national or subnational level, including case numbers, incidence, outbreaks, and EV serotype distributions. We excluded studies in which HFMD and herpangina cases were combined and could not be distinguished, given their overlapping EV aetiology. Further, we excluded articles published in languages other than English and those without English abstracts. Studies focusing primarily on virology (such as basic science, *in vitro* experiments, or animal models) or lacking clear relevance to HFMD epidemiology or aetiology were also excluded.

We identified additional information, including official websites that report surveillance data, through cross-referencing across relevant articles and review papers, particularly for countries with limited publicly available data.

**Figure 1:**
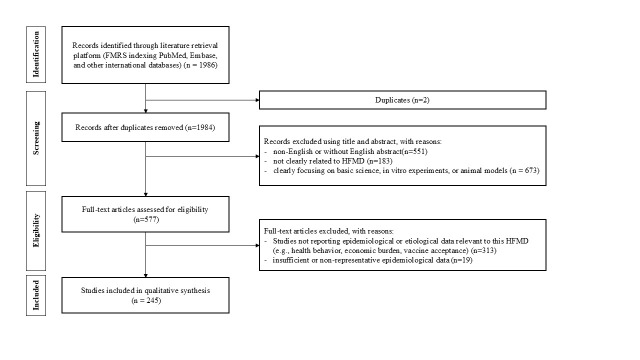
Flow diagram of study inclusion. The included studies were used for both the main synthesis and detailed country-level descriptions presented in Section S1 in the **Online Supplementary Document**. Additionally, contextual information extracted from reviews or articles was not included in this selection process.

We conducted the literature search in August 2025. Articles published since 2020 were searched using the term ‘hand, foot, and mouth disease’ or ‘HFMD’ to achieve broad coverage. A total of 1986 articles were identified from the platform. After screening and eligibility assessment, we included 245 full-text articles in the final synthesis (**Figure 1**).

### Data definitions and sources

We defined epidemiological data as population-level indicators that describe the occurrence and distribution of HFMD, including case counts, incidence, and reported outbreaks. Aetiological data referred to the distribution of EV serotypes causing HFMD based on laboratory-confirmed cases.

We categorised data sources into surveillance data and study-based data. Surveillance data refers to information derived from national monitoring systems, typically made publicly available on official websites or in surveillance reports. Study-based data referred to findings from published studies conducted in local institutions (such as hospitals or research institutions) that reported the distribution of HFMD-associated EV serotypes.

When publicly available, we prioritised surveillance data to characterise country-level epidemiological and aetiological patterns of HFMD. In some settings, particularly where official data were not publicly accessible, we obtained surveillance-derived data indirectly through published studies reporting national or subnational surveillance results.

In settings where surveillance data were unavailable, we drew evidence from published studies to provide descriptive summaries. For the same country and time period, data from multiple sources were synthesised to provide an overall description.

When multiple overlapping reports of the same outbreak or period were identified within a country, we cross-verified data, and the most comprehensive or most recent results were prioritised to avoid duplication.

Media reports were included only as supplementary evidence to support outbreak identification and were not used for epidemiological or aetiological inference.

### Data synthesis and analysis

Given the heterogeneity in surveillance systems and case reporting practices across countries, we adopted a qualitative synthesis approach to summarise the available evidence, focusing on the availability of surveillance, epidemiological trends, and aetiological patterns of HFMD.

To improve clarity in presenting aetiological patterns, we operationally defined a ‘major circulating serotype’ as one that accounts for more than 20% of all laboratory-confirmed HFMD cases in a given country or study period. This threshold was used for descriptive purposes only, including visualisation, and did not represent a strict epidemiological definition of dominance. No formal comparisons across countries, quantitative synthesis or meta-analysis were performed.

## RESULTS

### Surveillance and data availability of hand, foot, and mouth disease

Currently, no unified global surveillance system for HFMD has been established, and the availability of epidemiological and aetiological data varies substantially across countries. Historically, HFMD outbreaks have been predominantly reported in the Asia-Pacific region, where HFMD is commonly included in national notifiable disease surveillance systems, such as China [20,21], Vietnam [22,23], and Malaysia [24]. At the regional level, the WHO Western Pacific Regional Office established an HFMD reporting system in 2011; however, publicly available biweekly reports did not include laboratory diagnosis or EV serotyping [25,26], and were only accessible between 2015 and 2018 [27]. The Asia-Pacific Network for Enterovirus Surveillance, established in 2017 through collaborations between academic institutions and hospitals, currently includes a limited number of participating countries and regions [1].

**Figure 2:**
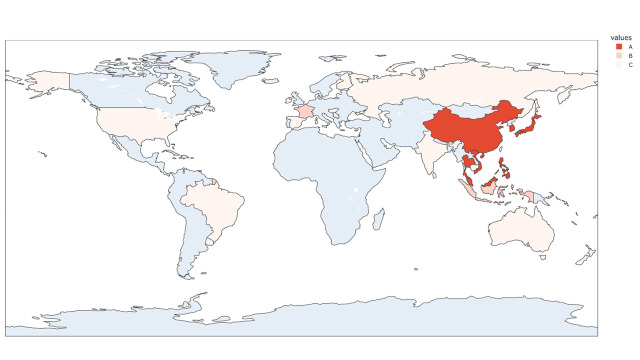
Global availability of surveillance systems and country-level data on HFMD. This map summarises the availability of country-level surveillance systems and data sources related to HFMD, categorised into three groups. **Panel A.** Countries where HFMD is monitored as a notifiable disease within routine national surveillance systems. **Panel B.** Countries with HFMD surveillance outside formal notifiable disease systems. **Panel C.** Countries where available evidence of HFMD is primarily derived from published studies. Countries shown in grey indicate the absence of publicly available surveillance or data. This figure highlights substantial geographic heterogeneity in surveillance systems and data availability, which constrains direct comparison of HFMD epidemiology across regions. *Country boundaries follow standard international definitions and may include overseas territories.

In Europe and North America, surveillance systems for non-polio EVs have been established, such as laboratory-based surveillance networks in Europe [28,29] and the National Enterovirus Surveillance System in the USA [1,30]. However, these systems are not syndrome-specific to HFMD and therefore may not fully reflect HFMD-specific epidemiological or aetiological patterns (**Figure 2**, **Table 1**).

**Table 1:** Country-level surveillance, major circulating EV serotypes and reported outbreaks of HFMD*

Country/region	Disease surveillance	Aetiological surveillance	Major circulating serotypes in recent years (after 2020)		Outbreaks reported since 2000
			**Serotypes**	**Data sources**	
China	National notifiable disease system (since 2008, publicly available) [20]	National sentinel hospital-based surveillance (since 2009, not publicly available) [21]	CV-A16; CV-A6; others increasing [31,32]	Published studies using surveillance data	2008–2015 [1,33]
Vietnam	National communicable disease surveillance system (since 2011, publicly available) [22,23]	Research-based surveillance (PI HCM; NIHE) [23]	Mainly EV-A71(2023) [34]	Published articles based on research groups	2005 [1,22]; 2011–2012 [33,35]; 2018 [33]; 2023 [34]
Malaysia	Mandatory notifiable disease (since 2006, publicly available) [24]	Mandatory notifiable disease (since 2006, not publicly available) [24]	CV-A6 (2023); CV-A16 (2024) [36].	Official report [36]	2000 [5,37]; 2003 [5,38]; 2006 [5,38]; 2007 [38]; 2018 [39,40]; 2022 [11]; 2025 [12,13]
Thailand	Notifiable disease (since 2001, not publicly available) [41]	A national surveillance program for HFMD to monitor EVs (not publicly available) [42]	Mainly CV-A6 [41,43]	Published studies	2012 [8,41,43,44]; 2017 [1,33,44]; 2019–2022 [43]; 2025 [14,15]
Japan	Notifiable infectious diseases (IDWR, publicly available) [45]	Notifiable infectious diseases (IASR, publicly available) [45,46]	CV-A16; CV-A6 [45,46]	Official Report (IASR) [46]	2000; 2003; 2006; 2010; 2011; 2013; 2015 [47]; 2017 [8]
Korea	National infectious disease surveillance system (since 2009, publicly available) [48–50]	A national surveillance of EV (since 1993, not publicly available) [33]	CV-A6 (2022) [51–53]	Published studies	2000 [5]; 2009–2012 [1,33]; 2022 [53]
Singapore	Once a legally notifiable disease [54,55]	NA	NA	NA	2000 [1,54]; 2008 [1]
The Philippines	Notifiable infectious diseases (not publicly available) [56]	Severe Enteroviral Disease (not publicly available) [56]	CV-A16 (2022) [57]	Published studies	2025 [16–18]
India	NA	NA	CV-A6; CV-A16 (2022) [58–62]	Published studies	2003 [63]; 2007 [64]; 2016 [65]; 2022 [60,66]
Indonesia	Required reported in hospitals (since 2025) [67]	Required reported in hospitals (since 2025) [67]	NA	NA	2016 [68]
France	PARI Medical Observatory (since 2017) [69–71]; Citywide sentinel surveillance system (since 2010) [72] (not publicly available)	PARI Medical Observatory (since 2017) [69–71]; Citywide sentinel surveillance system (since 2010) [72] (not publicly available)	CV-A6; CV-A16 (2021) [71]	Published studies	2021 [71]

CV-A16 – coxsackievirus A16, CV-A6 – coxsackievirus A6, EV-A71 – enterovirus A71, HFMD – hand, foot, and mouth disease, IASR – Infectious Agents Surveillance Report, IDWR – Infectious Disease Weekly Report, NA – not available, NIHE – National Institute of Hygiene and Epidemiology, PI HCM – Pasteur Institute in Ho Chi Minh City, PARI – Paediatrics and Ambulatory Research in Infectious Diseases

*This table summarises the availability of national surveillance systems for HFMD, major circulating EV serotypes and data sources, and outbreaks across countries included in this study. Surveillance systems vary substantially in scope and diagnostic depth, which may affect reported incidence and aetiological distribution. Serotype information reflects laboratory-confirmed cases where available and may not be directly comparable across countries due to differences in surveillance design and reporting completeness.

### Global epidemiology of hand, foot, and mouth disease

Based on publicly available data, the reported incidence of HFMD in 2023 varied substantially across countries, ranging from 118.71 per 100,000 population in China [73] to 409.6 per 100,000 population in Malaysia [36]. However, these estimates were not directly comparable due to differences in surveillance systems and reporting practices. In countries such as China [73] and Japan [45,74], HFMD incidence exhibited a biennial pattern, with higher incidence observed every other year.

During the COVID-19 pandemic, reported HFMD cases decreased markedly across most countries, particularly in 2020. This decrease has been widely associated with the implementation of non-pharmaceutical interventions [75–78]. Since 2021, the number of reported cases has gradually returned to levels comparable to the pre-pandemic period (2009–2019) [36,45,73,74,79]. Notably, Malaysia reported a sharp increase in HFMD cases and incidence in 2022 [36] (Section S1 in the **Online Supplementary Document**). However, these post-pandemic changes may not reflect true underlying epidemiological trends and may also be influenced by the relaxation of non-pharmaceutical interventions [75], which likely contributed to the decline in reported cases during the pandemic. Several studies reported a delay of approximately one to two months in the seasonal peak of HFMD epidemics following the COVID-19 period [75,80].

In recent years, an increasing number of HFMD outbreaks and epidemiological studies have been reported in countries outside the Asia-Pacific region, including Uruguay [81], Serbia [82], and Palestine [83] (**Table 1**; Section S1 in the **Online Supplementary Document**).

The absence of routine HFMD surveillance and limited data availability in many of these countries limited the ability to distinguish between true absence of transmission and under-reporting or under-detection. This may contribute to both surveillance gaps and epidemiological heterogeneity.

Although HFMD predominantly affects children under five years of age, cases have also been reported among neonates [84] and individuals aged six years and older [85,86]. Sporadic cases involving adolescents, adults, and pregnant women have been reported in multiple countries (Section S2 in the **Online Supplementary Document**). While these reports were not representative of population-level epidemiology, they may indicate potential changes in transmission dynamics or expanding susceptible populations.

### Major circulating enterovirus serotypes causing hand, foot, and mouth disease

Although several countries routinely conducted serotype surveillance for HFMD-associated EVs, much of the aetiological information was not publicly available; consequently, available data largely came from outbreak-driven investigations and published studies (**Table 1**), resulting in substantial variability in data sources across countries, with differences in data coverage and representativeness.

Prior to 2020, EV-A71 and CV-A16 were commonly reported as predominant EV serotypes associated with HFMD outbreaks in multiple Asian countries [33], often co-circulating with CV-A10 and CV-A6. Together, these four serotypes accounted for a large proportion of laboratory-confirmed HFMD cases reported in countries including China [87], Vietnam [23,88,89], Malaysia [39,40], Thailand [41,43], Japan [46], Korea [51,52], Singapore [90,91], and the Philippines [56] (Section S1 in the **Online Supplementary Document**). In India, CV-A6 and CV-A16 have consistently been reported as major circulating serotypes across multiple studies [64,92–95]. In China, especially following the introduction of EV-A71 vaccination, the proportion of HFMD cases caused by EV-A71, particularly severe cases and deaths, declined markedly, as indicated by multiple studies [20,87,96–98].

**Figure 3:**
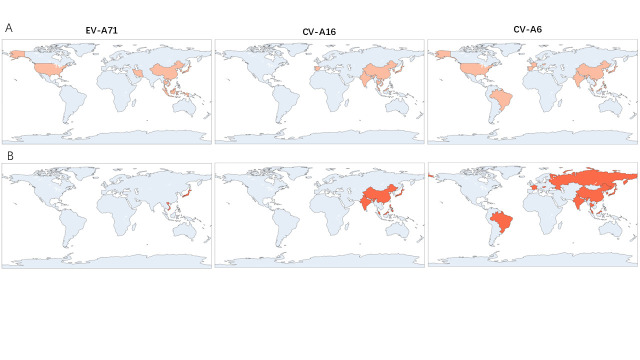
Global distribution of major circulating enterovirus serotypes causing HFMD before and after 2020. This figure illustrates changes in the geographic distribution of the three major EV serotypes associated with HFMD – EV-A71, CV-A16, and CV-A6 – before 2020 and since 2020, based on surveillance data and published studies. For visualisation purposes, a serotype was defined as major circulating when it accounted for ≥20% of laboratory-confirmed HFMD cases within a given country or outbreak. Serotypes detected at lower proportions were not displayed to avoid overinterpretation of sporadic or minor circulation. Countries shown in grey indicate the absence of publicly available aetiological data rather than the confirmed absence of HFMD transmission.

Outside the Asia-Pacific Region, aetiological data were primarily derived from outbreak-driven studies. These reports consistently identified CV-A6 as the major circulating serotype causing HFMD outbreaks reported in many countries, such as the USA [8,99], Brazil [100–102], France [69,72], England [103–105], Spain [106–108], and Hungary [109], with EV-A71 reported sporadically [1,104,110] (**Figure 3**, Panel A).

In the past five years (since 2020), CV-A6 has been increasingly reported as a major circulating serotype causing HFMD in multiple countries, including Thailand [41,43], Brazil [111], Hungary [112], Russia [113], and Cape Verde [114]. In contrast, CV-A16 has been reported intermittently as a major circulating serotype in countries such as China [31,32], Japan [46], Malaysia [36], Korea [53], the Philippines [57], and France [71]. In India, CV-A6 and CV-A16 have continued to co-circulate as the main HFMD-associated serotypes [58–62,115,116]. EV-A71 was reported less frequently in Japan [46] but remained associated with outbreaks in Vietnam in recent years [34] (**Figure 3**, Panel B). Notably, these patterns should be interpreted with caution, as changes over time may not necessarily reflect causal effects of the COVID-19 pandemic.

Several studies have reported that CV-A6 is frequently associated with atypical HFMD presentations [71,105]. Given that most HFMD cases were diagnosed based on clinical symptoms, differences in clinical presentation and diagnostic criteria may influence case detection and reporting.

In addition to the four major serotypes, sporadic HFMD cases and outbreaks caused by other EVs were reported, including CV-A4 [117,118], CV-A5 [7,119], and CV-A8 [120], as well as multiple CV-B [112,121–124] and echovirus serotypes [112,125–128]. Some studies also identified EV serotypes associated with severe HFMD cases, including those with central nervous system complications [100,125,129,130].

## DISCUSSION

This review synthesises available country-level evidence to characterise recent global epidemiological and aetiological patterns of HFMD over the past five years. The findings highlight substantial heterogeneity in reported cases, incidence, outbreaks, and major circulating serotypes of HFMD across countries, largely reflecting differences in surveillance coverage and data availability. In many settings, available evidence is primarily derived from outbreak-driven investigations and local studies, which may limit the comparability of findings and should be considered when interpreting the observed patterns. These findings underscore the importance of developing standardised surveillance and aetiological monitoring to advance the understanding of global HFMD epidemiology and aetiology.

The increasing number of reported HFMD cases and outbreaks in countries outside the traditionally recognised Asia-Pacific region suggests that HFMD transmission may be more geographically widespread than previously recognised. However, in the absence of standardised and systematic HFMD surveillance in many countries, it remains difficult to distinguish between actual changes in transmission dynamics and under-detection. In addition, the absence of publicly available data in some countries does not necessarily indicate the absence of HFMD; it may instead reflect limited data accessibility. These findings underscore the importance of expanding surveillance coverage and improving data comparability across countries and regions, particularly through targeted or sentinel surveillance in underrepresented settings.

Notably, reported HFMD cases decreased markedly during the COVID-19 pandemic and subsequently returned to levels comparable to, or, in some settings, exceeding those observed before the pandemic. These changes may not necessarily reflect true underlying epidemiological trends but may also be influenced by the implementation and relaxation of non-pharmaceutical interventions, as well as other factors such as changes in healthcare-seeking behaviour and the recovery of surveillance systems. Given the limited availability of studies on this topic, further research is needed to better understand the underlying drivers of these observed patterns.

With respect to aetiological patterns, observed changes suggest a shift in the circulation of EV serotypes in recent years. In this article, 2020 was used as a pragmatic time point to describe differences in major circulating serotypes between the preceding period and the most recent five years. Although this time point coincides with the onset of the COVID-19 pandemic, such temporal overlap does not imply a causal relationship. Additionally, available data suggested that major circulating serotypes may vary from year to year, and there is currently no conclusive evidence supporting a direct impact of the COVID-19 pandemic on aetiological patterns.

The observed shift from EV-A71 and CV-A16 dominance toward an increasing circulation of CV-A6 in many countries suggests a potential change in the global HFMD landscape. This pattern may have important implications, as CV-A6 has been more frequently associated with atypical clinical presentations of HFMD, which may contribute to underdiagnosis or misclassification in settings relying primarily on clinical diagnosis.

However, the apparent increase in CV-A6 may not necessarily reflect true underlying epidemiological changes and may also reflect improved recognition of atypical HFMD and potential publication bias. Additionally, heterogeneity in data sources may contribute to variability in observed aetiological patterns, as data in many countries are primarily derived from local studies rather than systematic surveillance.

Efforts to standardise surveillance and laboratory methods may enhance the comparability of HFMD data. The establishment of the European Non-Polio Enterovirus Network in 2021 represented an attempt to standardise data collection, monitor EV circulation, and estimate disease burden across Europe through standardised protocols [29,131]. Such initiatives highlighted the potential value of coordinated surveillance and unified diagnostic approaches for HFMD. Additionally, the continued detection of a broad range of EV serotypes associated with HFMD, including those linked to severe disease and central nervous system complications, further illustrates the dynamic and unpredictable nature of EV circulation.

The widespread deployment of monovalent EV-A71 vaccines has substantially reduced severe HFMD cases and mortality in some settings; however, the increasing predominance of non-EV-A71 serotypes, particularly CV-A6 and CV-A16, may suggest that monovalent vaccines have limited capacity to address the diversity of circulating serotypes. Emerging epidemiological evidence suggests that multivalent vaccine strategies may be considered to address the diversity of circulating serotypes. More relevant data are essential to inform vaccine strain selection, evaluate vaccine impact, and guide evidence-based policy decisions at both national and international levels.

Collectively, these findings indicated the importance of strengthening unified HFMD surveillance, improving standardisation in monitoring and diagnostics, and expanding aetiological monitoring to advance understanding of global HFMD epidemiology and support effective prevention and control strategies. Although this review aimed to synthesise global epidemiological and aetiological evidence on HFMD as comprehensively as possible, given the available data, several limitations should be acknowledged.

First, a substantial proportion of the included studies were outbreak-driven or localised, which may not fully represent population-level aetiological patterns of HFMD-associated EVs. This reflects the broader limitation of available data, as routine and standardised surveillance systems are lacking in many countries.

Second, language restrictions may have introduced bias. Although many excluded articles were likely published in Chinese and may therefore have limited impact on the overall findings, studies published exclusively in local languages and not indexed in major international databases may have been missed, potentially leading to underrepresentation of data from certain regions, particularly Africa and Latin America.

Third, due to limited availability of surveillance data and data gaps across countries, no formal cross-country comparisons or in-depth quantitative analyses were conducted. Future studies may help to address these limitations.

Despite these limitations, this study provides a country-level scoping synthesis of currently available evidence and highlights critical gaps in global HFMD data. Future studies based on population-level, standardised, longitudinal, and geographically representative data will be essential to advance the understanding of global HFMD epidemiology and aetiology, improve comparability across regions and support robust epidemiological analysis.

## CONCLUSIONS

This study provides a country-level synthesis of available evidence describing global epidemiological and aetiological patterns of HFMD. Although HFMD has historically been regarded as concentrated in the Asia-Pacific region, recent reports of emerging outbreaks from other regions suggest its transmission may be more geographically widespread than previously recognised. However, substantial gaps in surveillance and data availability limit the ability to draw definitive conclusions.

The increasing circulation of CV-A6, alongside continued circulation of CV-A16 and EV-A71 and the emergence of other serotypes, emphasises the dynamic nature of EV epidemiology. Importantly, much of the observed variation across countries may reflect differences in surveillance systems and data availability rather than true epidemiological differences.

Developing unified surveillance systems, improving diagnostic standardisation, and expanding aetiological monitoring are essential for enhancing data comparability, supporting accurate disease burden estimation, and informing evidence-based HFMD prevention and control strategies.

**Data availability:** All data analysed in this study were obtained from previously published articles and publicly available surveillance reports. All data sources were cited within the article and its **Online Supplementary Document**.

## Additional material


Online Supplementary Document
