# Supplementary material for: Global epidemiological and aetiological patterns of hand, foot, and mouth disease: a country-level scoping synthesis
Source: J Glob Health. 2026 Jul 17;16:04220. doi: 10.7189/jogh.16.04220 (PMC13377766; doi:10.7189/jogh.16.04220)
Supplement: Online Supplementary Document [file jogh-16-04220-s001.pdf]

Supplement to: Li Z, Han X, Ren Y. Global epidemiological and etiological patterns of hand, foot, and mouth disease: a country-level scoping synthesis. J Glob Health. 2026;16:04220.

Online Supplementary Materials

Table S1. Adherence to JoGH's GRABDROP guidelines items.

**JoGH guideline items**
1. Please list all papers published by each co-author in previous 3 years that were based on secondary analysis of a big data repository
None of the authors have published papers based on secondary analysis of large-scale data repositories in the past three years.
2. Please explain the key elements of your study design and the use of the available datasets that make your study an original scientific contribution
This study was designed as a country-level scoping synthesis to characterise the global epidemiological and etiological patterns of hand, foot, and mouth disease (HFMD) based on available global evidence.<br>By systematically collecting and synthesising data from heterogeneous sources(national surveillance systems and published studies), we provide a structured overview of HFMD epidemiology across countries. Detailed country-specific information is presented in the **Supplementary Materials** to support transparency and reproducibility.<br>A key element of our study is the focus on country-level mapping of evidence, which allows us to identify substantial heterogeneity in surveillance and data availability for both epidemiological and etiological patterns across regions. Our findings highlight that many countries lack systematic surveillance data or have very limited published evidence on HFMD epidemiology and serotype distributions.<br>The originality of this study lies in its integrated, country-level perspective, moving beyond traditional narrative reviews focused on pathogens, clinical features or vaccine development. Instead, it provides a comprehensive synthesis of available evidence and explicitly identifies critical data gaps. Additionally, changes in major circulating serotypes highlight the need for more comprehensive and accessible data, as well as standardised diagnostic and laboratory detection procedures.<br>Overall, our study provides a foundation for future research and underscore the importance of developing unified surveillance systems, standardizing diagnostic and reporting approaches, and improving global data comparability for HFMD.
3. Please list all publications that addressed similar research questions in the same dataset and indicate where you cited them in your paper
The following relevant review articles addressing similar research topics were cited in the Introduction section and Results of this manuscript.<br>Additional information, including data from official surveillance websites, was extracted from the following review papers to ensure comprehensiveness, particularly for countries with limited publicly available data (as described in the Methods section).<br>1. Huang, C.Y., S.B. Su, and K.T. Chen, A review of enterovirus-associated hand-foot and mouth disease: preventive strategies and the need for a global enterovirus surveillance

network. Pathog Glob Health, 2024. 118(7-8): p. 538-548.
2. Zhu, P., et al., Current status of hand-foot-and-mouth disease. J biomed sci, 2023. 30(1): p. 15.
3. Leung, A.K.C., et al., Hand, Foot, and Mouth Disease: A Narrative Review. Recent Adv Inflamm Allergy Drug Discov, 2022. 16(2): p. 77-95.
4. Puenpa, J, Wanlapakorn, N, Vongpunsawad, S, et al. The History of Enterovirus A71 Outbreaks and Molecular Epidemiology in the Asia-Pacific Region. J Biomed Sci. 2019; 26 (1): 75. doi: 10.1186/s12929-019-0573-2

To our knowledge, no previous studies have conducted a country-level synthesis using a comparable dataset or approach. Therefore, the present study represents a distinct scoping synthesis rather than a reanalysis of an existing dataset.

4. Please explain how you addressed multiple testing through an appropriately rigorous statistical threshold and indicate this in the methods section

This study was designed as a descriptive scoping review, focusing on summarizing and synthesizing available epidemiological and etiological data across countries. No formal statistical hypothesis testing or inferential comparisons were performed.
Therefore, issues related to multiple testing and adjustment for statistical significance thresholds are not applicable in this context. This has been clarified in the Methods section of the revised manuscript.

5. Please declare to what extent have AI chatbots been used in developing your paper and to which parts of the paper did they contribute

ChatGPT was used solely for language editing to improve clarity and readability. It did not contribute to study design, study selection, data synthesis, interpretation of results, or the formulation of conclusions. All content and scientific decisions were made by the authors.

Following material provided detail descriptions on country-specific epidemiology and etiology of hand, foot, and mouth disease (HFMD), and HFMD case reports in adults and pregnant in different countries. The summary on EV-associated HFMD outbreaks by years and countries since 2000 were detailed in **Table S2.**

Section 1 Descriptions on epidemiology and etiology of HFMD in countries

China

Since the largest-scale outbreak in Fuyang city, Anhui Province, in 2008, HFMD has emerged as a significant public health concern in China[1]. In 2008, HFMD was classified as a category III notifiable infectious disease and has since been monitored by a national surveillance system[1]. According to the Guidelines on prevention and control of HFMD(2009 version), throat swab or stool specimens were required to be collected from clinically diagnosed HFMD cases for laboratory serotype analysis[2].

Surveillance data from 2023 indicated a total of 1,673,481 reported cases of HFMD was, with an incidence of 118.71 per 100,000[3](**Figure S1**). Notably, a meta-analysis indicated that the recessive infection rate of HFMD-associated enterovirus (EV) among healthy individuals in China ranged from 4.59% to 44.12%[4]. Analysis has shown a sharp decrease in severe illness rate and mortality of HFMD across all age groups following the use of the EV-A71 vaccine, with reductions of 62.20% and 83.78%, respectively[5]. Additionally, the timing of the HFMD epidemic peak was delayed by 1-2 months[5]. However, HFMD continued to cause numerous local outbreaks, as Zhejiang Province had reported, a total of 47 HFMD and HA outbreaks occurred from 2021 to 2023[6].

In general, the proportions of each serotype in laboratory-confirmed cases[7] and outbreak cases[8] of HFMD in different years were shown in the **Figure S2** and **Figure S3**. A review on HFMD in China revealed that from 2008 to 2012, the predominant serotypes were EV-A71(50.56%) and CV-A16(28.88%) [9]. From 2013 to 2019, EV-A71, CV-A16, CV-A6 and CV-A10 co-circulated. During this period, the prevalence of CV-A6 and CV-A10 increased, while that of EV-A71 decreased[1,9]. CV-A16 and CV-A6 became the predominant serotypes particularly during 2016 and 2020[9]. Still, most severe and fatal cases were associated with EV-A71 infection, accounting for over 60% and 90% of such cases, respectively[2,10,11].

The serotypes causing HFMD also exhibited regional variation, differing among provinces and even cities. Studies indicated a continuously high CV-A16 infection rate in southern China from 2008 to 2014[12]. After a period of lower epidemic activity in 2015-2017, an upsurge of CV-A16 infection was observed in 2018-2019, likely resulting from the introduction(s) from Southeast Asia[12]. The causative serotypes in Yunnan Province

changed to non-EVA71/non-CVA16 enteroviruses from 2008 to 2019 [13], a trend also observed in Gansu Province from 2010 to 2021[14]. EV-A71 was the dominant serotype in Hubei Province during 2010–2019, especially in 2016[15]. CV-A16 was the main EV causing HFMD in Shanxi Province from 2009 to 2020, with an average proportion of 33.06%[16]. In Jiangxi Province, EV-A71 remained the dominant serotype causing severe HFMD until 2017, after which the proportion of other EVs significantly increased, accounting for 100% of cases in 2021 and 2023[17]. In Zhejiang province, there was a shift from CV-A16 in 2021 and 2022 to CV-A6 in 2023[6]. In Shiyan, Hubei Province, data from 2009 to 2019 suggested that CV-A6 may have been the most common serotype in hospitalized HFMD cases[18].

Molecular characteristic analysis of different EVs that published between 2020 and 2025 were conducted at the country, province and city levels. Analysis of EV-A71 were performed in Beijing[19] and Xiamen[20]; CV-A16 analyses were conducted nationwide[21,22] and in Beijing[23], Guangdong[24], Shanxi[25] and Shenyang[26]; CVA6 analyses were performed nationwide[27-29] and in Beijing[30-32], Hebei[33], Guangxi[34], Henan[35], and Linyi[36]; and the analysis of CV-A10 were conducted in Zhejiang[37], Shanghai[38], and Taiyuan[39].

In addition to four main serotypes of EV, cases caused by other EVs had also been reported: CV-A4 was reported in mainland China in 2011[40], and was identified in Jiangsu Province from 2016 to 2018[41]. An outbreak caused by CV-A4, with febrile illness and influenza-like illnesses as the main symptoms, was reported in Shandong Province in 2021[42]. Sparse HFMD cases caused by CV-A5 have been reported in China[40], and it was one of the major serotypes causing epidemics from 2016 to 2017 in Hubei Province[43]. There had also been reports of HFMD caused by CV-A8 in China[44].

Study also indicated that aforementioned EVs may also cause HFMD with central nervous system (CNS) complications. A study showed that the serotypes causing CNS complications were EV-A71 (40%), followed by CV-A4 (17%), CV-A2 (13%), CV-A10 (10%), CV-A6 (7%), and CV-A16 (4%)[45]. Another study identified the serotypes causing severe HFMD cases and deaths included CV-A2, CV-A4, CV-A6, CV-A9, and CV-A10[33]. Furthermore, research showed that CV-A1 may be related to severe HFMD cases[46]. Analyses also indicated that CVB were also important agent associated with HFMD[47], such as CV-B1[48], CV-B2[49], and CV-B4, a potential virus that could cause HFMD outbreak[50]. Studies had also shown that echovirus could cause HFMD, such as E3[33,51], E9[52], E11[33], and E30[53].

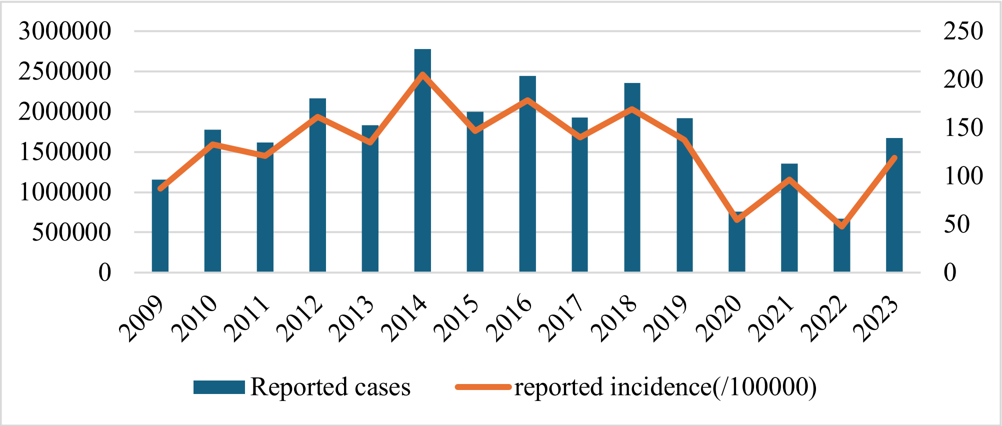

Figure S1. Reported cases and incidence rates of HFMD in China, 2009-2023[3]

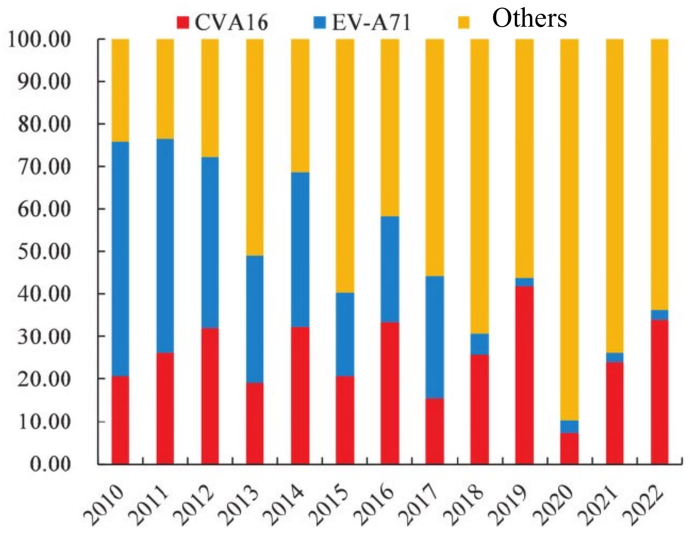

Figure S2. Serotype distributions of laboratory-confirmed HFMD in China, 2010-2022[7]

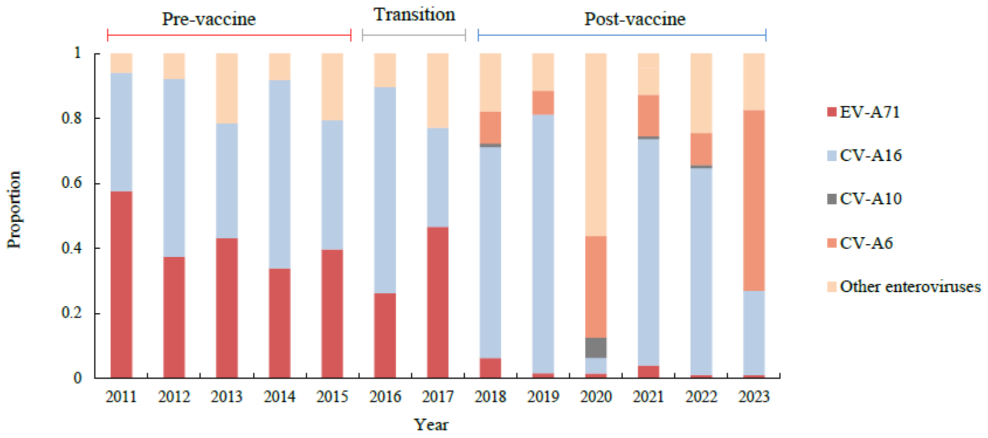

Figure S3. Serotype distributions in HFMD outbreak cases, 2011-2023[8]

The pre-vaccine time was from 2011 to 2015. The vaccine transition time was between 2016 and 2017. The post-vaccine time was from 2018 to 2023

Vietnam

In Vietnam, the first HFMD case was reported in 2003[54]. An outbreak caused by CV-A16 and EV-A71 occurred in 2005[55,56], followed by an increase in reported cases from 2007 to 2009[56]. The largest HFMD outbreak in Vietnam occurred from February 2011 to July 2012, resulting in 174,677 cases and 200 deaths upon hospital admission[54].

Since 2011, HFMD has been included in the national communicable disease surveillance system (Circular No. 48/2010/TT-BYT)[56,57]. The numbers of reported cases and the incidences of HFMD increased until 2018 (134,657 cases; incidence: 142.24/100,000) [58]. In 2020, the number of reported cases decreased to 83,789, with an incidence of 8.84/100,000[58](**Figure S4**).

Etiological surveillance in Vietnam was primarily set up by research groups at the Pasteur Institute in Ho Chi Minh city (PI HCM) and the National Institute of Hygiene and Epidemiology(NIHE) in Hanoi, beginning in 2005 and 2008, respectively[57].

The NIHE has conducted surveillance in the northern provinces[59]. Results indicated that the dominant serotypes in northern provinces of Vietnam between 2008 to 2018 were EV-A71 (31.7-35.04%), CV-A6 (26.57-28.4%), and CV-A16(20.84-22.4%)[57,60]. An analysis conducted between 2012 and 2017 in Hai Phong and Thai Binh yielded similar results, with the proportions of CV-A6, EV-A71, and CV-A16 being 32.5%, 26.9% and 19.3%, respectively[59]. A study showed that the CV-A6 (47.1%) and EV-A71(32.5%) were the main cause of HFMD in northern Vietnam in 2015-2016[54].

From 2012 to 2021, among all the hospitalized HFMD patients with identified EV detections, EV-A71 accounted for 49.06% of cases, followed by CV-A6(22.99%), CV-A10(9.94%), and CV-A16(7.06%)[61].

A study conducted by Oxford University Clinical Research Unit, Ho Chi Minh City between 2013 and 2015 indicated that the four dominant serotypes in Ho Chi Minh City were EV-A71 (26.2%), CV-A6 (17.8%), CV-A16 (11%), and CV-A10 (7.1%)[62]. Another analysis from 2015 to 2018 showed that EV-A71(28.3%), CV-A6(16.4%), CV-A16(11.5%) and CV-A10(7.9%) were the causative agents[63]. The molecular epidemiology of CV-A16 in southern Vietnam had also been reported[64].

A sudden increase in the prevalence of CV-A10 in 2016 and CV-A2 and CV-A4 in 2017[59]. And in 2018, a large-scale outbreak caused by EV-A71 infection occurred[65]. Additionally, a study on the infection of EV-A71, CV-A6, and CV-A16 among household contacts highlighted the need to consider the household as an additional target for HFMD intervention programs[66].

More recently, a HFMD outbreak occurred in 2023, with a total of 12,600 HFMD cases and 7 deaths reported in Vietnam[67]. A study conducted during this outbreak found that 98.8% of EV-positive samples were positive for EV-A71[67].

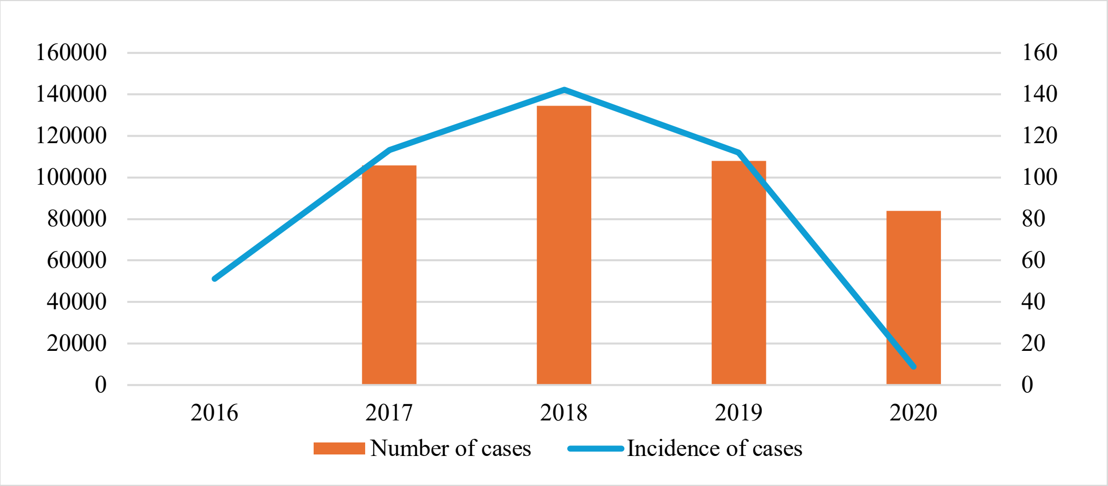

Figure S4. Reported cases and incidence rates of HFMD in Vietnam, 2016-2020[58]

Malaysia

After being first reported in 1997 in Malaysia, HFMD was made a mandatory notifiable disease in 2006 under the Prevention and Control of Infectious Diseases Act 1988 [Act 342] [68]. National incidences of HFMD from 2009 to 2024 were available from the Ministry of Health (**Figure S5**), and the main EVs causing HFMD in 2023 and 2024 were CV-A6 and CV-A16, respectively [69].

The first documented HFMD outbreak, which occurred in Sarawak (East Malaysia) in 1997 and subsequently spread to peninsular Malaysia, was caused by EV-A71 infection, affecting more than 4,000 children and 41 deaths[70,71]. Although CV-A16 was also isolated during that outbreak, only EV-A71 was identified in fatal cases[70]. Since then, EV-A71 epidemics have occurred every 2 to 3 years, in 2000, 2003, 2006, 2008/2009, and 2012[71]. In late 2000, another HFMD outbreak occurred in Malaysia, followed by a recurrence of an outbreak caused by EV-A71 in 2003[72]. An outbreak in late 2005 saw CV-A16 preceded the appearance of EV-A71, which was followed by a larger outbreak in Sarawak in early 2006[72]. In 2007, an outbreak of HFMD was only noted in peninsular Malaysia that was almost purely due to CV-A16[72]. Further epidemics of EV-A71 occurred in 2008, 2009 and 2012[71]. In an outbreak that occurred in peninsular Malaysia in 2010, both EV-A71 and CV-A16 were identified as the main serotypes[72].

During 2018, a major HFMD outbreak occurred across Malaysia with over 76,000 infected individuals[73]. A study from a teaching hospital in Kuala Lumpur showed that among all

EV-positive samples, the proportion of CV-A6, CV-A16, EV-A71, CV-A10, and CV-B3 were 44%, 40%, 10%, 2%, and 4%, respectively[73]. A study conducted during an outbreak in Beaufort, Sabah, in 2018 showed that 83.3% of samples were positive for CV-A16 and 16.7% for EV-A71[70].

Notably, in 2022, a HFMD outbreak in Malaysia was reported on the social media[74], followed by the dramatically increasing number of reported HFMD cases in 2022 (**Figure S5**)[69]. In 2025, news reports indicated another HFMD outbreak in Malaysia. The number of reported HFMD cases by late May reached 99,601, a 266% increase compared to the same period in the previous year[75,76]

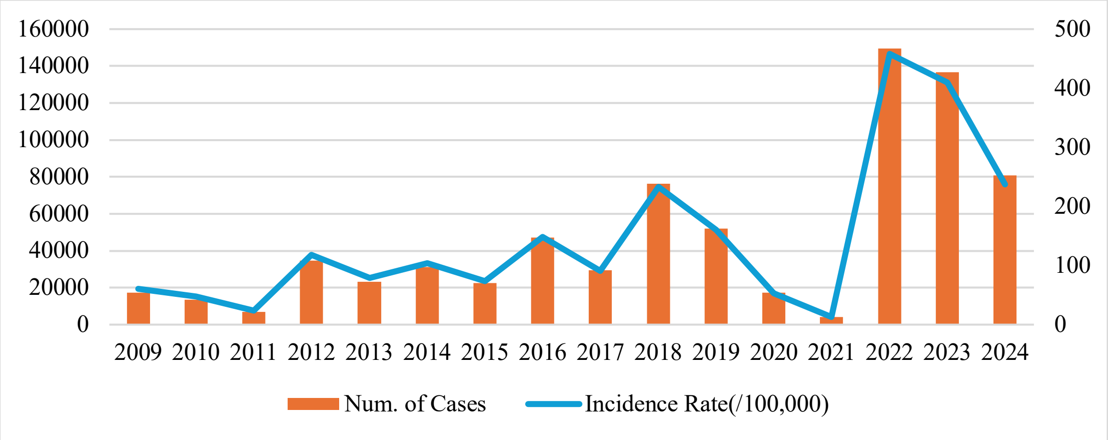

Figure S5. Reported cases and incidence rates of HFMD in Malaysia, 2009-2024[69]

Thailand

HFMD was first noticed in 2000 in Thailand and became a notifiable disease under the Ministry of Public Health (MOPH) in 2001[77].

The first outbreak was recorded in 2012 with 45,464 infected individuals, the largest in a decade[78]. Subsequent outbreaks occurred in 2016 and 2017[65,78]. Confirmed cases from 2011 to 2019, sourced from Bureau of Epidemiology (BoE) National Disease Surveillance (report 506) showed that there was a sharp peak in the number of HFMD cases during the rainy season of each year [78].

EV-A71 was first detected in Thailand in 2001, promoting the launch of a national surveillance program for HFMD to monitor EVs[79]. For the last few decades, EV-A71 and CV-A16 have been the major EV causing HFMD in Thailand[77]. CV-A6 was detected sporadically during 2008 and 2011[80]. In 2012, a large HFMD outbreak caused by CV-A6 was reported, with over 60% of affected children being less than three years of age[77,80]. Following that outbreak, CV-A6 co-circulated with EV-A71 and CV-A16[77], causing epidemics with a clear two-year recurrent cyclical pattern(outbreaks in 2012, 2014, 2016, and

2018)[77,80]. Another nationwide HFMD outbreak in 2017 was caused by EV-A71 and CV-A6[65,78]. More recently, a large outbreak due to CV-A6 occurred between January 2019 and October 2022[77,80]. Serotype analyses during this period showed that 23.7% of samples were positive for CV-A6, 3.7% for EV-A71, 3.4% for CV-A16, and 17.9% for other serotypes[80].

Etiological analysis of other EVs associated with HFMD, such as CV-A4[81], have also been conducted. A study using samples collected between 2000 and 2020 analyzed the molecular evolutionary dynamics of EVs causing HFMD, showing the distribution and proportion of EV genotypes and implying that EV continues to evolve, potentially causing large-scale outbreaks in susceptible populations[77].

According to online news, there were 48,154 reported HFMD cases reported nationwide between January and August, 2025, indicating an outbreak, with most cases occurring in children under five years old[82,83].

Japan

HFMD epidemics occur annually in Japan, typically in the summer season and primarily affecting children, but large-scale epidemics have occurred every two years since 2011[84].

HFMD is classified as a Category V notifiable infectious diseases in Japan, with approximately 3,000 pediatric sentinel sites nationwide reporting on a weekly basis[85]. The prevalences and incidences of HFMD at these sites have been published in the Infectious Disease Weekly Report (IDWR) since 1999 (**Figure S6**) [84-86]. And the data on causative serotypes of HFMD is available by the Infectious Agents Surveillance Report(IASR) [87].

According to these reports, the numbers of HFMD cases varies greatly from year to year[85]. In the decade from 2009 to 2019, the lowest number of cases was in 2016 (69,139 cases), and the highest was in 2019 (402,529 cases)[85,86]. In 2020, the number of cases was much lower than usual (18,364 cases) [85,86]. From 2021 to 2023, the number of reported cases returned to levels comparable to the 2009-2019 period[85]. In 2024, the number of reported HFMD cases has increased since the 15th week, reaching levels similar to pre-COVID 19 era and significantly exceeding the weekly average of the past five years[85].

In 2000 and 2003, major epidemics of HFMD were caused mainly by EV-A71; Smaller epidemics of HFMD continued until 2006, when EV-A71 and CV-A16 were mainly isolated[88]. In 2010, Japan again experienced a large epidemic of HFMD caused mainly by EV-A71 again[88]. Since then, large-scale HFMD outbreaks occurred every 2 years (in 2011, 2013, 2015, and 2017) in Japan, but the major causative EV gradually transferred to CV-A6[84,88].

According to the Infectious Agents Surveillance Report(IASR) [87].The main EVs isolated from HFMD patients in the last five years were CV-A16 and CV-A10 in 2020; CV-A6 and CV-A16 in 2021 and 2022; EV-A71 and CV-A6 in 2023; and CV-A6 and CV-A16 in 2024[85,87].

Additionally, case reports on the clinical features of HFMD caused by CV-A6 have been published[89,90], indicating a possibility associated to onychomadesis[90,91]. Sporadic HFMD cases caused by CV-A5 have also been reported in Japan[40].

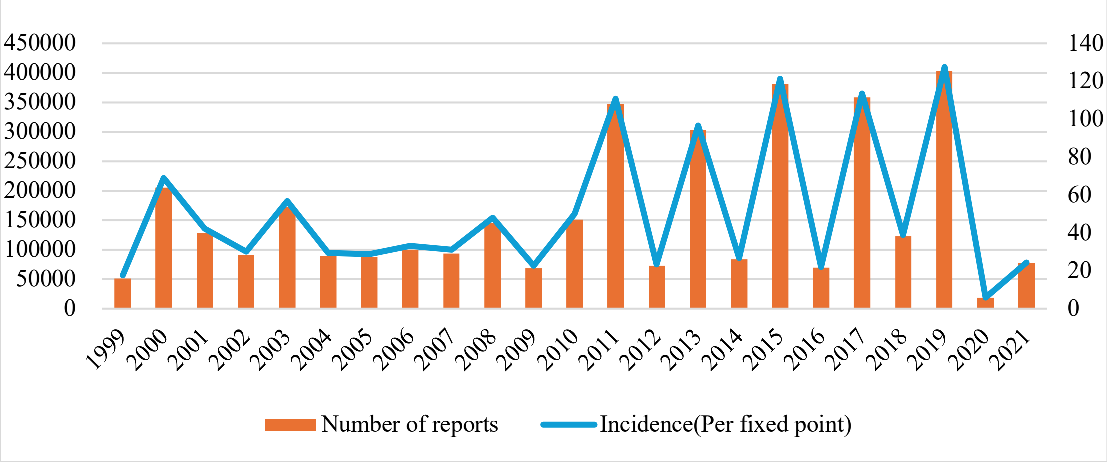

Figure S6. Reported cases and incidence rates of HFMD in Japan, 1999-2021[86]

Korea

The Korea Centers for Disease Control and Prevention launched a national surveillance of enterovirus since 1993[65]. HFMD was monitored through voluntary reporting by pediatricians starting in 2008 and was designated as a national infectious disease in 2009, requiring reporting through a surveillance system[92-94]. According to annual surveillance reports, data of reported HFMD cases are available from 2015 to the present[94]. There was a peak in reported cases in 2019 (1847 cases) [94]. The number of reported cases increased significantly starting in 2022 (from 12 cases in 2021 to 1,180 in 2022, 614 in 2023, and 1,044 in 2024)[94]. No HFMD cases with complication reported in 2020 or 2021[94].

A study used data from the South Korea Health Insurance Review and Assessment Service (HIRA) between 2011 and 2017 showed a total of 1,879,342 HFMD cases in children younger than six years old (8.4 of 100 persons in this age group), with most cases occurring in children under three[92].

EV71 outbreak in Korea was first documented in 2000[95] and HFMD outbreak caused by EV-A71 occurred during 2009 and 2012[55,65]. An analysis using data from Korea Enterovirus Surveillance System (KESS) from 2012 to 2019 identified 1,373 HFMD cases

caused by EVs, including 155 cases with complications[96]. Most cases were in children under five years old. Among HFMD cases, EV-A71 and CV-A6 were the main serotypes (28.9% and 20.4%), followed by CV-A16 (16.4%), CV-A10 (11.8%), and CV-A5(4.2%)[96]. For HFMD with complications, the main viruses were EV-A71 and E18(20.4% each), followed by E30(10.8%), CV-B5(9.7%), and E6(8.6%)[96]. A study in Gwangju showed that EV-A71 and CV-A16 were the main causes of HFMD (23.80% and 20.48%), followed by CV-A6 (19.43%), CV-A10(10.54%), CV-A5(5.12%) and CV-A4(2.41%) from 2011 to 2020[97].

In 2022, an HFMD epidemic occurred in Gwangju, mainly caused by CV-A6, which was the first epidemic since the COVID-19 pandemic began[98].

Singapore

In Singapore, a surveillance system for HFMD, based on notifications from child-care centers, was implemented since 1998[99]. Reporting of the disease became legally mandatory in 2000[99]. Data on reported cases and incidences were available from 2003 to 2018[100].

HFMD was first detected in 1970 in Singapore[99]. Outbreaks caused by CV-A16 and EV-A71 had been reported since then[99]. EV-A71 caused a large HFMD epidemic at the end of 2000(with about 3,800 reported cases)[99] and remain circulating in 2006 and 2008[101], while CV-A16 was the mainly circulating EV in 2005, 2007 and 2009[101]. In 2008, Singapore experienced its largest ever HFMD outbreak caused by EV-A71 and CV-A strains, resulting in 29,686 cases[55]. After late 2009, CV-A6 became the predominant serotype causing HFMD outbreaks[101]. A study of symptomatic paediatric HFMD cases from 2013 to 2018 identified CV-A6 as the major etiological agent(41.0%), followed by EV-A71 (7%), CV-A16 (3.0%), CV-A2 (1.0%) and CV-A10 (1.0%)[102]. Additionally, CV-A4 was reported to have caused illness in Singapore in 2008[40].

Currently, HFMD is not a legally notifiable disease in Singapore; however, educational institutions are required to notify the Ministry of Health (MOH) of any HFMD outbreaks[103].

The Philippines

HFMD is classified as an immediately notifiable disease in the Philippines; samples from suspected cases and their corresponding epidemiological data are collected through the Philippine Integrated Disease Surveillance and Response (PIDSR) for HFMD and Severe Enteroviral Disease[104].

A study that randomly selected and tested uncharacterized EVs from 2012 and 2013 showed an increase in the detection rate of CV-A6 among EV-positive HFMD cases, from 61.9% in 2012 to 88.1% in 2017[104]. The majority of the laboratory-confirmed HFMD cases caused by CV-A6 were young children with a median age of two years old[104]. A cluster of suspected HFMD cases was reported in a school in Balangao, Pangasinan Province, in September 2022[105]. Active case-finding revealed that the majority of cases (78%) were six years old, with CV-A16 being the main serotype(67%)[105].

News reports indicated an HFMD outbreak in the Philippines from January to June 2025, with numbers significantly higher than in the same period of previous year[106-108].

India

India identified the first HFMD case in 2003, with EV-A71 being the causative serotype[109,110]. The first HFMD outbreak was reported in 2003 among children in Calicut(northern Kerala state, southwestern India)[111]. Significant morbidity and mortality were reported in children with EV-A71 infection from Uttar Pradesh (UP) from 2004 to 2006[112]. In 2007, the first large-scale HFMD outbreak occurred in West Bengal[109]. Since then, many small-scale outbreaks have been repeatedly reported from different locations in northeast India, often based on clinical description without laboratory-confirmed reports[109,113].

HFMD cases with CV-A16 and CV-A6 as the predominant viruses were reported from Southern and Eastern parts of India in 2009–2010 [110]. From 2013 to 2015, multiple outbreaks were reported from Karnataka, Gujarat, Maharashtra, Assam and the Andaman Islands; CV-A16 was the predominant causative agent, but CV-A6 and EV-A71 were also detected[110]. A study in Dibrugarh and Tezpur in 2014 verified the circulation of CV-A6 and CV-A16 among HFMD cases[109]. Subsequently, another large-scale outbreak of HFMD was reported in Ladakh in 2016[114].

A study conducted in southern India from 2015 to 2017 showed that CV-A6, CV-A16, and CV-A10 accounted for 64%, 15% and 1% of all samples, respectively[115]. Samples collected at a Tertiary Care Hospital in 2016 and 2017 verified that the circulating serotypes were CV-A6 (31.6%) and EV-A71 (21.6%)[116]. Another study during an outbreak in 2017 identified CV-A6 (75%) and CV-A16(25%) as the associated serotypes[117]. Samples collected from HFMD cases from 2017 to 2018 in Western India revealed the presence of CV-A16(57.7%), CV-A6 (40.8%), and E1 (1.4%)[118].

An HFMD outbreak occurred in India in 2022[119,120]. Studies indicated the main serotypes were CV-A6 and CV-A16[110,121,122]. A multicenter study showed that 94.13% of cases

were positive for IgM antibodies to CV-A6, CV-A16 or EV-A71, with antibodies against CV-A6 being the most commonly detected[122]. Multiple studies conducted during HFMD outbreaks confirmed the main circulation of CV-A16 and CV-A6[110,112]. A study in Navi Mumbai, Maharashtra showed 56.5% of samples collected from children were positive for CV-A6, 15.9% for CV-A16, and 1.4% for CV-A4; one case of co-infection (CV-A16 and CV-A6) was also reported[121]. Another study in Karnataka showed that CV-A16 (68.4％) and CV-A6(31.6％) were the main causative agent of HFMD[123]. HFMD cases continued to be reported in 2023, with most cases occurring in children under three years old[124].

Overall, no surveillance system for HFMD was specified in India. However, HFMD cases have been continually reported in India, with CV-A6 and CV-A16 being the most common serotypes. Molecular analysis of CV-A16[111,125] and CV-A6[126] were also conducted. The HFMD outbreak in 2022 caused serious concern due to the potential risk of future epidemics and the possibility of multiple co-occurring disorders[122,127]. Additionally, case reports related to HFMD during outbreaks[128] and possible complications, such as onycholysis[129], had also been reported.

Indonesia

There is no specific surveillance system for HFMD in Indonesia. HFMD cases were reported in Yogyakarta in 2014, although the exact number of patients was not identified[130]. An HFMD outbreak occurred in Banjarmasin, Indonesia in 2016 and reported 18 cases positive for EV-A71 serotype [130]. According to research from the Virology Laboratory Centre for Biomedical and Basic Health Technology at the National Institute of Health Research Centre, among 48 suspected cases from 2008 to 2021, 26 (54%) were caused by EVs, including 3 cases of EV-A71 (6.25%)[130].

A serosurvey conducted in Bandung city and the West Bandung region between 2022 and 2023 showed a high seroprevalence of EV-A71 IgG, indicating the circulation of EV-A71[130].

In 2025, HFMD cases caused by EV-A71 are required to be reported in hospitals under the categories of “acute meningoencephalitis syndrome” or “acute rash syndrome” in the guideline Technical Guidelines for Sentinel Surveillance of Emerging Infectious Diseases in Hospitals[131].

The United States

There is no specific surveillance system for HFMD in the United States. As introduced in the main text, the National Enterovirus Surveillance System (NESS) was designated to collects basic data on specimens positive for enterovirus or human parechovirus in the United States since the 1960s[55,132]. NESS showed that the most frequently detected EVs was CV-B4 in 2023 and EV-D68 in 2024[133], but these cannot fully reflect HFMD-specific etiological patterns.

HFMD caused by CV-A6 was reported in Boston in 2012[134]. A study recorded a total of 27,996 HFMD cases from 2005 to 2013 in California, without etiological data[135].

An outbreak of neurologic disease caused by EV-A71 occurred in Colorado during 2003 and 2005[55]. Again in 2018, Children's Hospital Colorado noted another outbreak, during which EV-A71 was later identified in 58% of those with enterovirus neurological disease, 93% had findings suggestive of meningitis, 72% showed evidence of encephalitis, and 23% met the case definition of acute flaccid myelitis[136]. In the same year, an atypical outbreak of HFMD among college students at a mid-size university in the mid-Atlantic U.S. was reported, with all tested samples positive for CV-A6[137]. Another HFMD outbreak reported at a university occurred between mid-September and mid-October, 2023[138]. As diagnoses were made clinically, no etiological data was available[138].

Brazil

Although notification is not mandatory in Brazil, frequent outbreaks associated with CV-A6 are reported, particularly in settings such as daycare centers and schools[139].

Notably, CV-A6 was rarely associated with HFMD in Brazil until 2016[139]. A previous study identified CV-A16 and CV-A6 as the main serotypes of HFMD in approximately 75 stool samples typed from eight Brazilian states between 2009 and 2016[140]. During 2018, Brazil experienced widespread HFMD outbreaks caused by CV-A6[141]. Another study in Belém, in the Amazon region, found CV-A6 in about 82% of blood samples from HFMD patients in 2019[139,140]. A study reported on HFMD outbreaks in Sao Paulo State during 2021 identified a total of 4,718 related cases, with 92.98% of cases being children aged 1-4 years old and a seropositive rate of 27.5% for CV-A6[142].

In addition to CV-A6, other EV serotypes have also been reported to be associated with HFMD in Brazil, including coxsackievirus types CV-A1, CV-A2, CV-A4, CV-A5, CV-A9, CV-A16, CV-A24, and CV-B5; echovirus types E11, E13, E14, E18, and E25; and enterovirus types EV-A71, EV-C99, and EV-C116[139]. Case reports about sequalae of HFMD, such as onychomadesis, have also been documented[143].

France

A nationwide network, the PARI Medical Observatory, was established in May 2017 with the support of several French pediatric associations[144,145]. Electronic medical records (EMR) from primary care pediatricians are automatically extracted and sent to the Observatory for processing[144,145]. The network is designated for the prospective surveillance of common pediatric infectious diseases in outpatient settings, including HFMD/herpangina (HA)[146].

A citywide sentinel surveillance system of HFMD/HA was set up in 2010, with results showed that the predominant serotypes were CV-A10 (39.9%) and CV-A6 (28%), followed by CV-A16 (17.5%) and EV-A71 (6.3%); the predominant clinical presentation was HA (63.8%) and this was frequently associated with clinical signs of HFMD (48%)[147].

Between 2010 and 2018, CV-A6 was the predominant serotypes (50.4%) among the 1,946 EV-associated HFMD cases reported during the ambulatory surveillance[144]. The detection rates varied from 24% in 2013 to 64% in 2015[144]. Of the cases, 22% of children had typical HFMD and 72% had atypical HFMD[144]. It was worth noting that nationwide HFMD sentinel surveillance was interrupted between 2015 and 2017[144].

A study reported eight cases of EV-A71 infection diagnosed in Marseille in 2019 and 2020, indicating the circulation of this virus[148]. An HFMD outbreak occurred in France in 2021, with 3,403 cases reported (47% higher than in 2018-2019)[146]. An analysis conducted during this outbreak showed that CV-A6 was the predominant EV(49.5%), followed by CV-A16 (22.1%) and CV-A5 (11.1%)[146]. Results indicated that CV-A6 and CV-A16 were mostly associated with atypical HFMD (79.8% and 61.9%, respectively), while CV-A5 was more frequently associated with herpangina alone (52.4%)[146].

England

In England, 952 HFMD cases that mainly attributed to CV-A16 were reported in 1994[149]. The number of laboratory-confirmed EV-A71 detections reported by health authorities showed a biennial pattern and a slight increasing trend between 2006 and 2013, with peak years reporting no more than 50 detections; in contrast, the number of laboratory-confirmed CV-A6 detections showed a clear increasing trend over time, surpassing 150 detections per year since 2014[150,151].

Studies showed that seroprevalences of EV-A71 and CV-A6 increased from 32% and 54% in infants aged 6-11 months to more than 75% by 10 years of age[151]. Atypical HFMD was linked to newly emerging variants of CV-A6 infection in children in Edinburgh in 2014[152]. Additionally, the molecular epidemiology of CV-A6 has been analyzed[153].

A study using daily HFMD consultation rates from 2017 to 2022 provided an epidemiological overview of disease trends, with the mean weekly consultation rate for HFMD being 1.53 per 100,000 registered patients[154].

A HFMD case caused by CV-A16 was reported in a 13-month-old girl and her 5-year-pld brother, who developed rashes one week after returning from a family holiday to Kerala in May 2022[155].

Hungary

Since HFMD is not a notifiable disease in Hungary, no surveillance data on its incidence is available[156]. However, results from studies showed the infections of HFMD. A study reporting on EV types detected in clinical specimens from 2010 to 2018 showed 43% of HFMD cases were CV-A6 infections[156]. Another study of samples collected from 2020 to 2024 in patients with symptoms such as HFMD, encephalitis, or meningitis who tested positive for EVs showed that 52.80% of samples were CV-A6, others included CV-A10(4.80%), CV-B4(2.40%), CV-B5(2.40%), CV-A16(0.80%), E6(4.00%), E11(0.80%), E18(0.80%), E9(0.70%), and EV-A71(2.40%)[157].

Spain

In Spain, there is surveillance system for EVs, and the Spanish National Centre for Microbiology (CNM) has increasingly received more samples from local outbreaks and sporadic cases of HFMD that occurred in different regions, both for virological diagnosis and/or type identification since 2010[158].

During 2008 and 2012, several outbreaks and sporadic cases that associated to typical HFMD, atypical HFMD, or onychomadesis presentations occurred in different regions of Spain[158,159]. The research using samples collected from 2006 to 2020 through poliovirus and non-polio enterovirus (NPEV) surveillance showed that typical and atypical HFMD had a proportion of 58.4% and 38% of all cases, respectively, with the most frequent EV detected being CV-A6 (60.9%)[159]. CV-A16 was the only causative serotype of HFMD in 2010, however, by the end of the year and during 2011, CV-A6 became predominant. In 2012, both CV-A6 and CV-A16 circulated[158]. A study in Barcelona showed that CV-A6 was the most frequently detected EV among the EV-A viruses (70%) and was associated with atypical HFMD (78%)[160].

Additionally, cases caused with neurologic disease caused by EV-A71 were also reported in Spain[161,162].

Other countries

Italy Although there is no formal surveillance system for HFMD in Italy, a study showed that from 2012 to 2022, internet search activities followed a constant trend with peaks in November-December and in June[163]. Additionally, a case report described a 3.9-year-old boy developed Guillain–Barré syndrome (GBS) ten days after the diagnosis of HFMD[164]. And some HFMD cases may present in unusual locations, as described in some case reports[165].

Uruguay An HFMD outbreak occurred between April and June 2019, affecting at least 179 children in Paysandú Province[166]. A study showed that most samples collected during this outbreak were positive for CVA6 and CVA16, with only one cases associated with CVA10[166]. In September 2022, two children were hospitalized with presumptive rhombencephalitis, exhibiting symptoms of HFMD[167].

Cape Verde In July 2023, an alert was received from the Integrated Disease Surveillance and Response Service (SVIR) in Cape Verde regarding an increasing number of cases with fever and cutaneous rash, mostly in children under two years old. A study indicated that this may be the first-ever reported outbreak of CV-A6 associated with atypical HFMD in children in the country[168].

Russia A study in the Ural Federal District and Western Siberia of Russia in 2023 showed that HFMD was the prevailing clinical manifestation (36.7%) of all diseases caused by non-polio enteroviruses (NPEVs), and CV-A6 was the most common serotype in HFMD cases (47.87%)[169].

Serbia In Belgrade, Serbia, a total of 20 HFMD outbreaks were registered between 2015 and 2019. The year 2016 had a higher number of epidemics, involving 102 children from kindergartens[170]. Another study indicated that most cases (72.29%) occurred in children under six years old, but no etiological data were indicated[171].

Mexico Several apparent HFMD cases among children in a daycare center in Mexico City in 2012 suggested the existence of this disease[172]. A laboratory-confirmed case of HFMD in a 5-year-old male in Merida City in the Yucatan Peninsula of Mexico, with EV-A71 as the

etiological agent, was reported, providing the first peer-reviewed evidence of HFMD in the countries[172].

Iran A cross-sectional study conducted in 2015 showed 56.7% of samples collected from children aged 6 months to over 31 years old were positive for EV-A71 neutralizing antibody, with the presence of the antibody increasing with age[173].

Ireland An increase in the number of inpatient admissions for HFMD was noted in a hospital in 2023 compared to 2012 and 2013[174].

Palestine An outbreak occurred in the Jenin district in 2024, and a characterization of CV-A16 was analyzed using samples collected during the outbreak[175].

Peru A case of previously healthy five-year-old girl who presented with HFMD and subsequently developed onychomadesis one month later was reported[176].

Saudi Arabia A case was reported of a 6-year-old girl who present with HFMD symptoms again, six months after a similar presentation, although recurrence of HFMD is rare in children[177].

Argentina A case was reported of a 4-year-old boy infected with HFMD caused by CVA6[178].

Canada A case of typical HFMD was reported in 2020[179].

Greece CV-A4 was reported to have caused illness in Greece in 2009–2010[40].

Table S2. Summary on EV-associated HFMD outbreaks by years and countries since 2000

Years	Countries	Etiology	Results
2000[55,99]	Singapore	EV-A71	Total cases: 3,790
2000[180]	Korea	EV-A71	N.A.
2000[88]	Japan	EV-A71	N.A.
2000[180] [71]	Malaysia	EV-A71	Total cases: <169
2003[72,180]	Malaysia	EV-A71 and CV-A16	Total cases: <107
2003[88]	Japan	EV-A71	N.A.
2005[55,56]	Vietnam	EV-A71 and CV-A16	Total cases: 764<br>Deaths: 3
2003[111]	India	EV-A71	N.A.
2006[72,180]	Malaysia	EV-A71 and CV-A16	Total cases: 2,599
2006[88]	Japan	EV-A71 and CV-A16	N.A.
2006[180]	Brunei	EV-A71	Total cases: <1,681
2007[72]	Malaysia	CV-A16	Cases: 5,380
2007[109]	India	N.A.	N.A.
2008-2015[55,65]	China	EV-A71 and CV-A16	Total cases: 12,256,102<br>Severe: 123,261<br>Deaths: 3,322
2008[55]	Singapore	EV-A71, CV-A6, CV-A10	Total cases: 29,686<br>Deaths: 47
2008[134]	Finland	CV-A6 and CV-A10	Total cases:317
2009-2012[55,65]	Korea	EV-A71	Total cases: 7,385<br>Deaths: 5
2010[88]	Japan	EV-A71	N.A.
2010[147]	France	CV-A6 and CV-A10	N.A.
2011[88]	Japan	CV-A6	N.A.
2012[65]	Cambodia	EV-A71	Severe cases: 59<br>Deaths: 52
2011-2012[54,65]	Vietnam	EV-A71	Total cases: 174,677<br>Deaths: 200
2012-2013[65]	Australia	EV-A71	Total cases: 119<br>Deaths: 4
2012[134]<br>[77,78,80]	Thailand	CV-A6	Total cases: 45,464
2010-2012[158,159]	Spain	EV-A71, CV-A16, CV-A6	N.A.
2012-2013[134]	New Zealand	CV-A6	Cases: 98
2013[88]	Japan	CV-A6	N.A.
2013[113]	India	N.A.	Total cases: 47
2013[55]	Russian	EV-A71	Total cases: 78
2015[88]	Japan	CV-A6	N.A.
2016[114]	India	N.A.	Cases: 465

2016[170]	Serbia	N.A.	Cases: 102
2016[130]	Indonesia	N.A.	N.A.
2017[55,65,78]	Thailand	EV-A71 and CV-A6	Total cases: 70,733<br>Deaths: 3
2017[134]	Japan	CV-A6	Total cases: 6,173
2018[65]	Vietnam	EV-A71	Total cases: >53,000<br>Deaths: 6
2018[70,73]	Malaysia	CV-A6 and CV-A16	Total cases: >76,000
2018[141]	Brazil	CV-A6	N.A.
2018[137]	The U.S.	CV-A6	Cases: 138
2019[166]	Uruguay	CV-A6 and CV-A16	Cases: 179
2021[55]	France	CV-A6	Total cases: 3,403
2021[55]	Brazil	CV-A6	Total cases: 315
2019-2022[80]	Thailand	CV-A6	N.A.
2022[55]	India	CV-A16, EV-A71, CV-A6	Total cases: 196
2022[98]	Korea	CV-A6	Total cases: 277
2022[74]	Malaysia	N.A.	Outbreak 29,000+
2023[67]	Vietnam	EV-A71	Cases:12,600<br>Death: 200
2023[138]	The U.S.	N.A.	Cases: 60
2023[168]	Cape Verde	CV-A6	N.A.
2024[175]	Palestine	CV-A16	N.A.
2025[75,76]	Malaysia	N.A.	Cases: 99,000+
2025[82,83]	Thailand	N.A.	Cases: 48,000+
2025[106-108]	The Philippines	N.A.	N.A.

1. *this table was updated on the basis of a previous review[55,65,134,180]
2. *N.A. not available.

Section 2 Case reports of HFMD in adolescents and adults, and pregnant women

Infections in adults were often linked to contact with infected children[181]. These case reports emphasized the importance of considering HFMD in the differential diagnosis for adults presenting with unexplained cutaneous lesions and oral symptoms[182].In Japan, a 19-year-old female patient who had contact with a 3-year-old child two days before symptom onset was clinically diagnosed with HFMD after laboratory tests ruled out syphilis[183].

Case reports of HFMD in adults have documented in Japan[184], Vietnam[185], Indonesia[186], the United States[181,187-191], Canda[192], Italy[193-195], Germany[196], Netherlands[197], Denmark[198], Austria[199], Argentina[200], and Lebanon[201]. Cases with atypical symptoms have been reported in France(cases with)[202], Switzerland[203], and Portugal[182,204,205];

Some cases have involved complications and sequala after HFMD, such as optic Neuritis[206], secondary hypogonadism[207], conjunctivitis[208], unilateral acute idiopathic maculopathy[192,198,209,210], onycholysis and onychomadesis[188], maculopathy and retinopathy[199], encephalomyelitis[185], Guillain-Barré syndrome (GBS)[205], longitudinally extensive transverse myelitis[191];

These cases can be caused by EV-A71[182,185], CV-A4[184], CV-A10[193], CV-A6, which often related to atypical HFMD[195,197,211], and CV-B1 to CV-B6[200].

There are few reports on EV infections during pregnancy due to their low prevalence in the population, but such infections may cause severe neonatal complications[212].

Reports of HFMD during pregnancy[213] have been documented in Italy from 2010 to 2016, Although the role of EVs as causative agents of congenital defects remains uncertain, these cases of unfavorable outcomes suggest a need for caution and careful monitoring of pregnant women with HFMD throughout their gestation. Congenital CV-A6 infection may occur if a woman exhibits HFMD symptoms during the perinatal period[214]. Furthermore, infection with CV-A6 in late pregnancy can lead to fetal myocarditis and meningoencephalitis, with perinatal infection potentially resulting in fulminating multisystem involvement and death[215]. A female patient who was 28-weeks pregnant was diagnosed with HFMD after EV71 was detected in a serum virus antibody test, with a complication of pseudomembranous conjunctivitis[216]. Another case reported a woman who developed HFMD on the second day after spontaneous delivery[217]. A case was reported of an 18-year-old primigravida diagnosed with HFMD at 34 weeks of gestation[212].

Reference

1 Fu X, Wan Z, Li Y, Hu Y, Jin X, Zhang C. National Epidemiology and Evolutionary History of Four Hand, Foot and Mouth Disease-Related Enteroviruses in China from 2008 to 2016. Virol sin. 2020;35:21-33.

2 Ren MR, Cui JZ, Nie TR, Liu FF, Sun JL, Zhang YW, et al. [Epidemiological characteristics of severe cases of hand, foot, and mouth disease in China, 2008-2018]. Zhonghua Liu Xing Bing Xue Za Zhi. 2020;41:1802-7.

3 CDC C. Overview of the National Notifiable Infectious Disease Epidemic Situation in 2023. 2023. Available: https://www.chinacdc.cn/jksj/jksj01/202410/t20241010_301346.html. Accessed.

4 Zhou YJ, Niu XD, Ding YQ, Qian Z, Zhao BL. Prevalence of recessive infection of pathogens of hand, foot, and mouth disease in healthy people in China: A meta-analysis. Medicine. 2021;100:e24855.

5 Hong J, Liu F, Qi H, Tu W, Ward MP, Ren M, et al. Changing epidemiology of hand, foot, and mouth disease in China, 2013-2019: a population-based study. Lancet Reg Health West Pac. 2022;20:100370.

6 Chen Y, Sun W, Ren X, Gu X, Song K, Wang P, et al. Characteristics and timeliness of intervention in 47 school-based enterovirus outbreaks in Zhejiang Province, China. Front Public Health. 2025;13:1559637.

7 Yanzhe L, Yang S, Fengfeng L, Fan D, Ruohan S, Xin M, et al. Epidemiological characteristics of hand, foot and mouth disease caused by coxsackievirus A16 in China, 2010-2022. Journal of Tropical Diseases and Parasitology. 2025;23:65-9+97.

8 Liu F, Yi Y, Song Y, Zhang X, Xie T, Liu Y, et al. Epidemiology of hand, foot, and mouth disease outbreaks before and during availability of EV-A71 vaccine in China's mainland: analysis of outbreak surveillance data from 2011 to 2023. Lancet Reg Health West Pac. 2025;59:101603.

9 Zhang J, Li XH, Li XF, Shang X. [Etiology and epidemiology of hand, foot and mouth disease in China]. Zhonghua Liu Xing Bing Xue Za Zhi. 2022;43:771-83.

10 Cui JZ, Nie TR, Ren MR, Liu FF, Li Y, Wang LP, et al. [Epidemiological characteristics of fatal cases of hand, foot, and mouth disease in children under 5 years old in China, 2008-2018]. Zhonghua Liu Xing Bing Xue Za Zhi. 2020;41:1041-6.

11 Gao F, Tang BC, Jiang XL, Yin SY, Chang ZR, Qin Y, et al. [Epidemiological characteristics and trend of mortality on hand, foot and mouth disease in China, 2008-2022]. Zhonghua Liu Xing Bing Xue Za Zhi. 2024;45:1626-32.

12 Yi L, Zeng H, Zheng H, Peng J, Guo X, Liu L, et al. Molecular surveillance of coxsackievirus A16 in southern China, 2008-2019. Arch virol. 2021;166:1653-9.

13 Jiang L, Jiang H, Tian X, Xia X, Huang T. Epidemiological characteristics of hand, foot, and mouth disease in Yunnan Province, China, 2008-2019. BMC Infect Dis. 2021;21:751.

14 Jianjun Y, Yan F, Shu L, Xinfeng L, Li S, Xiaoshu Z, et al. Surveillance and model prediction of hand, foot and mouth disease in Gansu, 2010—2021. Disease Surveillance. 2024;39:318-23.

15 Wang W, Rosenberg MW, Chen H, Gong S, Yang M, Deng D. Epidemiological characteristics and spatiotemporal patterns of hand, foot, and mouth disease in Hubei, China from 2009 to 2019. PLoS One. 2023;18:e0287539.
16 Ren H, Liu Y, Wang XC, Li MC, Quan DC, Rao HX, et al. [Epidemiological characteristics and Spatial-temporal clustering of hand, foot and mouth disease in Shanxi province, 2009-2020]. Zhonghua Liu Xing Bing Xue Za Zhi. 2022;43:1753-60.
17 Yuan J, Cheng-feng Z, Huan-hong P, Tian-chen Z, Hai-long L, Yuan W, et al. Analysis of the epidemiological characteristics and trend changes of severe hand, foot, and mouth disease in Jiangxi Province from 2008 to 2023. Modern Preventive Medicine. 2024;51:4373-8.
18 Li JF, Zhang CJ, Li YW, Li C, Zhang SC, Wang SS, et al. Coxsackievirus A6 was the most common enterovirus serotype causing hand, foot, and mouth disease in Shiyan City, central China. World J Clin Cases. 2022;10:11358-70.
19 Li J, Liang Z, Huo D, Yang Y, Li R, Jia L, et al. Molecular epidemiology and phylodynamic analysis of enterovirus 71 in Beijing, China, 2009-2019. Virol J. 2023;20:256.
20 Chen M, He S, Yan Q, Zhang J, Li C, Su X, et al. Sporadic hand, foot, and mouth disease cases associated with non-C4 enterovirus 71 strains in Xiamen, China, from 2009 to 2018. Arch virol. 2021;166:2263-6.
21 Zhou Y, Van Tan L, Luo K, Liao Q, Wang L, Qiu Q, et al. Genetic Variation of Multiple Serotypes of Enteroviruses Associated with Hand, Foot and Mouth Disease in Southern China. Virol sin. 2021;36:61-74.
22 Han Z, Song Y, Xiao J, Jiang L, Huang W, Wei H, et al. Genomic epidemiology of coxsackievirus A16 in mainland of China, 2000-18. Virus Evol. 2020;6:veaa084.
23 Li R, Lin C, Dong S, Li J, Liang Z, Yang Y, et al. Phylogenetics and phylogeographic characteristics of coxsackievirus A16 in hand foot and mouth disease and herpangina cases collected in Beijing, China from 2019 to 2021. J med virol. 2023;95:e28991.
24 Zeng H, Zeng B, Yi L, Qu L, Cao J, Yang F, et al. The Emergence of Coxsackievirus A16 Subgenotype B1c: A Key Driver of the Hand, Foot, and Mouth Disease Epidemic in Guangdong, China. Viruses. 2025;17:-.
25 Guo J, Cao Z, Liu H, Xu J, Zhao L, Gao L, et al. Epidemiology of hand, foot, and mouth disease and the genetic characteristics of Coxsackievirus A16 in Taiyuan, Shanxi, China from 2010 to 2021. Front Cell Infect Microbiol. 2022;12:1040414.
26 Li F, Zhang Q, Xiao J, Chen H, Cong S, Chen L, et al. Epidemiology of Hand, Foot, and Mouth Disease and Genetic Characterization of Coxsackievirus A16 in Shenyang, Liaoning Province, China, 2013-2023. Viruses. 2024;16:-.
27 Chen Y, Chen S, Shen Y, Li Z, Li X, Zhang Y, et al. Molecular Evolutionary Dynamics of Coxsackievirus A6 Causing Hand, Foot, and Mouth Disease From 2021 to 2023 in China: Genomic Epidemiology Study. JMIR Public Health Surveill. 2024;10:e59604.
28 Zhao TS, Du J, Sun DP, Zhu QR, Chen LY, Ye C, et al. A review and meta-analysis of the epidemiology and clinical presentation of coxsackievirus A6 causing

hand-foot-mouth disease in China and global implications. Rev med virol. 2020;30:e2087.
29 Lu H, Xiao J, Wang W, Yan D, Ji T, Yang Q, et al. Evolutionary Diversity of Coxsackievirus A6 Causing Severe Hand, Foot, and Mouth Disease - China, 2012-2023. China CDC Wkly. 2024;6:442-9.
30 Cui Y, Yang YN, Zheng RR, Xie MZ, Zhang WX, Chen LY, et al. Epidemiological characteristics of hand, foot, and mouth disease clusters during 2016-2020 in Beijing, China. J med virol. 2022;94:4934-43.
31 Zhang M, Chen X, Wang W, Li Q, Xie Z. Genetic characteristics of Coxsackievirus A6 from children with hand, foot and mouth disease in Beijing, China, 2017-2019. Infect genet evol. 2022;106:105378.
32 Yu F, Zhu R, Jia L, Song Q, Deng J, Liu L, et al. Sub-genotype change and recombination of coxsackievirus A6s may be the cause of it being the predominant pathogen for HFMD in children in Beijing, as revealed by analysis of complete genome sequences. Int j infect dis. 2020;99:156-62.
33 Yu QL, Liu YY, Zhao WN, Su T, Xie Y, Zhang WW, et al. [Epidemiological and pathogenic characteristics of cases with severe and fatal hand, foot, and mouth disease caused by other enterovirus in Hebei province, 2013-2017]. Zhonghua Liu Xing Bing Xue Za Zhi. 2020;41:1054-7.
34 Ju Y, Tan Z, Huang H, Chen M, Tan Y, Zhang C, et al. Clinical and epidemiological characteristics of Coxsackievirus A6- and Enterovirus 71-associated clinical stage 2 and 3 severe hand, foot, and mouth disease in Guangxi, Southern China, 2017. J infection. 2020;80:121-42.
35 Chen S, Chen Y, Ji W, Wang F, Zhang X, Jin Y, et al. Emerging concerns of atypical hand foot and mouth disease caused by recombinant Coxsackievirus A6 variants in Henan, China. J med virol. 2023;95:e29316.
36 Chen J, You P, Chen X, Li H, Zhang N, Zhang G, et al. Genetic Characteristics and Phylogenetic Analysis of Coxsackievirus A6 Isolated in Linyi, China, 2022-2023. Jpn j infect dis. 2024;77:311-6.
37 Sun Y, Cai J, Mao H, Gong L, Chen Y, Yan H, et al. Epidemiology of hand, foot and mouth disease and genomic surveillance of coxsackievirus A10 circulating in Zhejiang Province, China during 2017 to 2022. J clin virol. 2023;166:105552.
38 Wang J, Liu J, Fang F, Wu J, Ji T, Yang Y, et al. Genomic surveillance of coxsackievirus A10 reveals genetic features and recent appearance of genogroup D in Shanghai, China, 2016-2020. Virol sin. 2022;37:177-86.
39 Wang J, Liu H, Cao Z, Xu J, Guo J, Zhao L, et al. Epidemiology of Hand, Foot, and Mouth Disease and Genetic Evolutionary Characteristics of Coxsackievirus A10 in Taiyuan City, Shanxi Province from 2016 to 2020. Viruses. 2023;15:694.
40 Gao F, Bian LL, Chen L, Zhou YP, Li GF, Mao QY, et al. A cross-sectional seroepidemiology study of seven major enteroviruses causing HFMD in Guangdong, China. J infection. 2021;83:119-45.
41 Guo WP, Chen GQ, Xie GC, Du LY, Tang Q. Mosaic genome of Human Coxsackievirus A4 associated with herpangina and HFMD in Yancheng, China, 2016 and 2018. Int j infect dis. 2020;96:538-40.

42 Li J, Ni N, Cui Y, Zong S, Yao X, Hu T, et al. An outbreak of a novel recombinant Coxsackievirus A4 in a kindergarten, Shandong province, China, 2021. Emerg Microbes Infect. 2022;11:2207-10.
43 Yu Y, Luo Z, Jin W, Mai J, Qian S, Lu J, et al. Emergence of a novel recombinant of CV-A5 in HFMD epidemics in Xiangyang, China. BMC Med Genomics. 2021;14:279.
44 Wang DY, Song Y, Han ZZ, Xiao JB, Lu HH, Yan DM, et al. [Genetic characterization analysis of the whole genome sequence of Coxsackievirus A8 associated with hand, foot and mouth disease in China]. Zhonghua Liu Xing Bing Xue Za Zhi. 2021;42:1487-92.
45 Li Y, Yang J, Liang L, Wang K, Turtle L, Li P, et al. Clinical characteristics and severity of hand, foot, and mouth disease by virus serotype: A prospective hospital-based cohort study. PLoS Negl Trop Dis. 2025;19:e0013039.
46 Feng CZ, Zhang M, Xu DH, Guo W, Sun H, Yang ZQ, et al. Complete Genome Analysis of A New Strain of Coxsackievirus A1 Associated with Severe HFMD in Yunnan, China. Biomed environ sci. 2022;35:248-53.
47 Liu Y, Chen J, Zhang M, Guo W, Feng C, Liu J, et al. Coxsackievirus B: The important agent of hand, foot, and mouth disease. J med virol. 2023;95:e28669.
48 Xu DH, Zhang M, Chen JW, Feng CZ, Liu YH, Chu ZY, et al. Molecular Characterization of Coxsackievirus B1 Strains Isolated from Patients with Hand Foot and Mouth Disease in Yunnan, Southwest China. Biomed environ sci. 2024;37:543-8.
49 Zhang M, Xu D, Feng C, Guo W, Fei C, Sun H, et al. Isolation and characterization of a novel clade of coxsackievirus B2 associated with hand, foot, and mouth disease in Southwest China. J med virol. 2022;94:2598-606.
50 Xiao J, Wang J, Zhang Y, Sun D, Lu H, Han Z, et al. Coxsackievirus B4: an underestimated pathogen associated with a hand, foot, and mouth disease outbreak. Arch virol. 2021;166:2225-34.
51 Chen J, Chu Z, Zhang M, Liu Y, Feng C, Li L, et al. Molecular characterization of a novel clade echovirus 3 isolated from patients with hand-foot-and-mouth disease in southwest China. J med virol. 2023;95:e29202.
52 Zhang M, Guo W, Xu D, Feng C, Bao G, Sun H, et al. Molecular characterization of echovirus 9 strains isolated from hand-foot-and-mouth disease in Kunming, Yunnan Province, China. Sci Rep. 2022;12:2293.
53 Zhang M, He D, Liu Y, Gong Y, Dong W, Chen Y, et al. Complete genome analysis of echovirus 30 strains isolated from hand-foot-and-mouth disease in Yunnan province, China. Virol J. 2023;20:215.
54 Chu ST, Kobayashi K, Bi X, Ishizaki A, Tran TT, Phung TTB, et al. Newly emerged enterovirus-A71 C4 sublineage may be more virulent than B5 in the 2015-2016 hand-foot-and-mouth disease outbreak in northern Vietnam. Sci Rep. 2020;10:159.
55 Huang CY, Su SB, Chen KT. A review of enterovirus-associated hand-foot and mouth disease: preventive strategies and the need for a global enterovirus surveillance network. Pathog Glob Health. 2024;118:538-48.

56 Donato C, Hoi IT, Hoa NT, Hoa TM, Van Duyet L, Dieu Ngan TT, et al. Genetic characterization of Enterovirus 71 strains circulating in Vietnam in 2012. Virology. 2016;495:1-9.
57 Hoa-Tran TN ea. Non-EV-A71 enteroviruses associated with hand, foot and mouth diseases among children aged under 5 years in Northern provinces, Vietnam, 2008-2018. Vietnam Journal of Preventive Medicine. 2018;28:15-28.
58 Yearbook of Health Statistics 2019-2020. 2025. Available: https://moh.gov.vn/thong-ke-y-te?p_p_id=useryearbook_WAR_yearbookserviceportlet&p_p_lifecycle=0&p_p_state=normal&p_p_mode=view&p_p_col_id=row-0-column-2&p_p_col_count=1&_useryearbook_WAR_yearbookserviceportlet_jspPage=%2Fhtml%2Fyearbook%2Fuser%2Fview.jsp&_useryearbook_WAR_yearbookserviceportlet_yearbookFileEntryId=1009. Accessed.
59 Hoa-Tran TN, Nguyen AT, Dao ATH, Kataoka C, Ta HTT, Nguyen HTV, et al. Genetic characterization of VP1 of coxsackieviruses A2, A4, and A10 associated with hand, foot, and mouth disease in Vietnam in 2012-2017: endemic circulation and emergence of new HFMD-causing lineages. Arch virol. 2020;165:823-34.
60 Hoa-Tran TN, Dao ATH, Nguyen AT, Kataoka C, Takemura T, Pham CH, et al. Coxsackieviruses A6 and A16 associated with hand, foot, and mouth disease in Vietnam, 2008-2017: Essential information for rational vaccine design. Vaccine. 2020;38:8273-85.
61 Romanenkova NI, Nguyen TTT, Golitsyna LN, Ponomareva NV, Rozaeva NR, Kanaeva OI, et al. Enterovirus 71-Associated Infection in South Vietnam: Vaccination Is a Real Solution. Vaccines (Basel). 2023;11:-.
62 Nhu L, Nhan L, Khanh T, Ny N, Van H, Hong N, et al. HAND, FOOT AND MOUTH DISEASE IN SOUTHERN VIETNAM DURING 2015 – 2021. Int J Infect Dis. 2023;130:S110-s1.
63 Lau EHY, Nhan LNT, Khanh TH, Hong NTT, Van HMT, Nhu LNT, et al. Clinical, etiological and epidemiological investigations of hand, foot and mouth disease in southern Vietnam during 2015 – 2018. PLOS Neglected Tropical Diseases. 2020;14.
64 Nhu LNT, Nhan LNT, Anh NT, Hong NTT, Van HMT, Thanh TT, et al. Coxsackievirus A16 in Southern Vietnam. Front Microbiol. 2021;12:689658.
65 Puenpa J, Wanlapakorn N, Vongpunsawad S, Poovorawan Y. The History of Enterovirus A71 Outbreaks and Molecular Epidemiology in the Asia-Pacific Region. J biomed sci. 2019;26:75.
66 Hoang CQ, Nguyen HD, Ho NX, Vu THT, Pham TTM, Nguyen KT, et al. Incidence of Infection of Enterovirus 71 and Coxsackieviruses A6 and A16 among Household Contacts of Index Cases in Dong Thap Province, Southern Vietnam. Biomed res int. 2020;2020:9850351.
67 Chau NVV, Thuong TC, Hung NT, Hong NTT, Quy DT, Thien TB, et al. Emerging Enterovirus A71 Subgenogroup B5 Causing Severe Hand, Foot, and Mouth Disease, Vietnam, 2023. Emerg infect dis. 2024;30:363-7.

68 Cox VM, Jahis R, Abu Bakar RS, Yusof NM, Pilay KPV, Chem YK, et al. Spatio-temporal dynamics of hand, foot and mouth disease in Malaysia, 2009-2019. PLoS Negl Trop Dis. 2025;19:e0013174.
69 Penerbitan Utama KKM (Main Publications of MOH). 2025. Available: https://www.moh.gov.my/index.php/pages/view/58?mid=19. Accessed.
70 Fong SY, Mori D, Rundi C, Yap JF, Jikal M, Latip A, et al. A five-year retrospective study on the epidemiology of hand, foot and mouth disease in Sabah, Malaysia. Sci Rep. 2021;11:17814.
71 NikNadia N, Sam IC, Rampal S, WanNorAmalina W, NurAtifah G, Verasahib K, et al. Cyclical Patterns of Hand, Foot and Mouth Disease Caused by Enterovirus A71 in Malaysia. PLoS Negl Trop Dis. 2016;10:e0004562.
72 Chua KB, Kasri AR. Hand foot and mouth disease due to enterovirus 71 in Malaysia. Virol sin. 2011;26:221-8.
73 Lee MHP, Chong YM, Tay CG, Koh MT, Chem YK, Noordin N, et al. Detection of enteroviruses during a 2018 hand, foot and mouth disease outbreak in Malaysia. Trop biomed. 2021;38:150-3.
74 Almost 29,000 HFMD cases recorded nationwide so far this year. 2022. Available: https://www.thestar.com.my/news/nation/2022/05/14/almost-29000-hfmd-cases-recorded-nationwide-so-far-this-year. Accessed.
75 Malaysia Catat Kenaikan Kasus Flu Singapura atau HFMD, Kebanyakan Serang Anak di Bawah 6 Tahun. 2025. Available: https://www.liputan6.com/health/read/6041942/malaysia-catat-kenaikan-kasus-flu-singapura-atau-hfmd-kebanyakan-serang-anak-di-bawah-6-tahun. Accessed.
76 Penang remains vigilant as HFMD cases decline. 2025. Available: https://www.thestar.com.my/metro/metro-news/2025/08/18/penang-remains-vigilant-as-hfmd-cases-decline. Accessed.
77 Noisumdaeng P, Puthavathana P. Molecular evolutionary dynamics of enterovirus A71, coxsackievirus A16 and coxsackievirus A6 causing hand, foot and mouth disease in Thailand, 2000-2022. Sci Rep. 2023;13:17359.
78 Verma S, Razzaque MA, Sangtongdee U, Arpnikanondt C, Tassaneetrithep B, Arthan D, et al. Hand, Foot, and Mouth Disease in Thailand: A Comprehensive Modelling of Epidemic Dynamics. Comput math method m. 2021;2021:6697522.
79 Puenpa J, Suwannakarn K, Chansaenroj J, Auphimai C, Wanlapakorn N, Vongpunsawad S, et al. Genetic diversity and evolution of enterovirus A71 subgenogroup C1 from children with hand, foot, and mouth disease in Thailand. Arch virol. 2021;166:2209-16.
80 Puenpa J, Saengdao N, Khanarat N, Korkong S, Chansaenroj J, Yorsaeng R, et al. Evolutionary and Genetic Recombination Analyses of Coxsackievirus A6 Variants Associated with Hand, Foot, and Mouth Disease Outbreaks in Thailand between 2019 and 2022. Viruses. 2022;15:73.
81 Puenpa J, Korkong S, Vichaiwattana P, Poovorawan Y. Genetic diversity and spread of recombinant coxsackievirus A4 in hand, foot, and mouth disease cases in Bangkok, Thailand: 2017-2023. Sci Rep. 2024;14:26902.

82 Thailand warns of hand, foot and mouth disease outbreak. 2025. Available: https://www.nationthailand.com/blogs/health-wellness/40051897. Accessed.

83 Hand, foot and mouth disease surges in young Thai children, cases up 65.7% in eight months. 2025. Available: https://www.thestar.com.my/aseanplus/aseanplus-news/2025/09/07/hand-foot-and-mouth-disease-surges-in-young-thai-children-cases-up-657-in-eight-months. Accessed.

84 Yoshida K, Fujimoto T, Muramatsu M, Shimizu H. Prediction of hand, foot, and mouth disease epidemics in Japan using a long short-term memory approach. PLoS One. 2022;17:e0271820.

85 IDWR 2024 年第 27 号 < 注目すべき感染症 > 手足口病 (IDWR No. 27 of 2024 <Infectious Diseases of Notable >). 2025. Available: https://id-info.jihs.go.jp/surveillance/idwr/idwr/2024/27/article/index.html. Accessed.

86 感染症発生動向調査年別一覧表 (Survey of Infectious Disease Outbreak Trends by Year). 2025. Available: https://www.niid.go.jp/niid/ja/ydata/10748-ydata2020.html. Accessed.

87 IASR 速報グラフ ウイルス(手足口病由来ウイルス)(IASR Bulletin Graph Virus). 2025. Available: https://id-info.jihs.go.jp/surveillance/iasr/graph/iasrgv/index.html. Accessed.

88 Takechi M, Fukushima W, Nakano T, Inui M, Ohfuji S, Kase T, et al. Nationwide Survey of Pediatric Inpatients With Hand, Foot, and Mouth Disease, Herpangina, and Associated Complications During an Epidemic Period in Japan: Estimated Number of Hospitalized Patients and Factors Associated With Severe Cases. J epidemiol. 2019;29:354-62.

89 Naomiya K, Ito T, Saito A, Igarashi T, Nakayama T, Katayama K, et al. Clinical Features and Characteristics of Hand, Foot, and Mouth Disease Caused by Recent Coxsackievirus A6: Five Cases in Japan from 2019 to 2022. Infect dis rep. 2024;16:587-92.

90 Takenaka K, Kawasaki K, Fukumoto T, Sakai K, Oka M. Coxsackievirus A6 infection presenting with different clinical phenotypes of hand-foot-and-mouth disease in two brothers at the same time. Eur j dermatol. 2020;30:62-3.

91 Takanosu T. Hand, foot and mouth disease-induced onychomadesis. BMJ Case Rep. 2024;17:e262621.

92 Baek S, Park S, Park HK, Chun BC. The epidemiological characteristics and spatio-temporal analysis of childhood hand, foot and mouth disease in Korea, 2011-2017. PLoS One. 2020;15:e0227803.

93 Kim BI, Achangwa C, Cho S, Ahn J, Won J, Do H, et al. The Hand, Foot, and Mouth Disease Sentinel Surveillance System in South Korea: Retrospective Evaluation Study. JMIR Public Health Surveill. 2024;10:e59446.

94 감염병감시연보 (Annual Report of Infectious Disease Surveillance). 2025. Available: https://www.kdca.go.kr/contents.es?mid=a20106000000. Accessed.

95 Won YJ, Kang LH, Lee AR, Paik B, Kim H, Lee SG, et al. Sequence analysis of the first B5 subgenogroup strain of enterovirus 71 isolated in Korea. J microbiol. 2020;58:422-9.

96 Kang HJ, Yoon Y, Lee YP, Kim HJ, Lee DY, Lee JW, et al. A Different Epidemiology of Enterovirus A and Enterovirus B Co-circulating in Korea, 2012-2019. J pediat inf dis soc. 2021;10:398-407.
97 Kim MJ, Lee JE, Kim KG, Park DW, Cho SJ, Kim TS, et al. Long-term sentinel surveillance of enteroviruses in Gwangju, South Korea, 2011-2020. Sci Rep. 2023;13:2798.
98 Lee JE, Kim MJ, Lim MH, Han SJ, Kim JY, Kim SH, et al. Epidemiological and Genetic Characterization of Coxsackievirus A6-Associated Hand, Foot, and Mouth Disease in Gwangju, South Korea, in 2022. Viruses. 2024;16:476.
99 Chan K, Goh K, Chong C, Teo E, Lau G, Ling A. Epidemic Hand, Foot and Mouth Disease Caused by Human Enterovirus 71, Singapore. Emerg infect dis. 2003;9:78-85.
100 Communicable Diseases Surveillance in Singapore. 2025. Available: https://www.cda.gov.sg/resources/. Accessed.
101 Kua JA, Pang J. The epidemiological risk factors of hand, foot, mouth disease among children in Singapore: A retrospective case-control study. PLoS One. 2020;15:e0236711.
102 Min N, Ong YHB, Han AX, Ho SX, Yen EWP, Ban KHK, et al. An epidemiological surveillance of hand foot and mouth disease in paediatric patients and in community: A Singapore retrospective cohort study, 2013-2018. PLoS Negl Trop Dis. 2021;15:e0008885.
103 Infectious Disease Notification (MOH_Singapore). 2025. Available: https://www.moh.gov.sg/seeking-healthcare/overview-of-diseases/communicable-diseases/infectious-disease-notification. Accessed.
104 Foronda JLM, Jiao M, Climacosa FMM, Oshitani H, Apostol LNG. Epidemiological and molecular characterization of Coxsackievirus A6 causing hand, foot, and mouth disease in the Philippines, 2012-2017. Infect genet evol. 2023;114:105498.
105 Victori EC, Ventura RJC, Blanco MZC, Pamintuan RP, Magpantay RL, Lonogan KB. School outbreak of hand, foot and mouth disease in Balungao, Pangasinan Province, Philippines, October 2022. West pac surveill re. 2023;14:1-5.
106 Klase sa elementarya kag tanan nga Day Care centers sa EB Magalona, suspendido sang 2 ka adlaw bangod sa mataas nga Hand, Foot, and Mouth Disease cases. 2025. Available: https://bacolod.bomboradyo.com/klase-sa-elementarya-kag-tanan-nga-day-care-centers-sa-eb-magalona-suspendido-sang-2-ka-adlaw-bangod-sa-mataas-nga-hand-foot-and-mouth-disease-cases/. Accessed.
107 HFMD cases in Iloilo balloon to 190%, children most affected. 2025. Available: https://www.panaynews.net/hfmd-cases-in-iloilo-balloon-to-190-children-most-affected/. Accessed.
108 2.5K kaso ng HFMD dumagsa(2.5K cases of HFMD spill over). 2025. Available: https://www.philstar.com/pilipino-star-ngayon/bansa/2025/08/31/2469319/25k-kaso-ng-hfmd-dumagsa. Accessed.

109 Borkakoty B, Das M, Jakharia A, Bhattacharya C, Bora C, Baruah PJ, et al. Hand, foot and mouth disease caused by Coxsackie viruses A6 and A16 in Assam, Northeast India: A need for surveillance. Indian j dermatol ve. 2020;86:105.
110 Chavan NA, Lavania M, Shinde P, Sahay R, Joshi M, Yadav PD, et al. The 2022 outbreak and the pathobiology of the coxsackie virus [hand foot and mouth disease] in India. Infect genet evol. 2023;111:105432.
111 Tikute S, Sonawane S, Shete A, Kumar A, Yadav S, Yadav PD, et al. Whole-genome sequencing and phylogenetic analysis of coxsackievirus-A16 strains causing hand, foot and mouth disease (HFMD) in India. Microb Genom. 2023;9:001130.
112 Diwate S, Yadav PD, Yadav J, Yadav AK. Enterovirus Coxsackie A16 Detected in Hand, Foot, and Mouth Disease Outbreak Among Children in Western Uttar Pradesh, India, May to June 2022. Asia-pac j public he. 2024;36:143-5.
113 Kashyap S, Verma GK. Hand-foot-mouth disease: outbreak in Shimla. Indian pediatr. 2014;51:155.
114 Sm K, Peecher J, L W, R C, Rehana K, T H, et al. Largest outbreak of hand, foot and mouth disease (HFMD) in India at an altitude of 3524 meters (11,562 ft.) in Leh, Union Territory (UT) of Ladakh, India 2016. J Hum Virol Retrovirol. 2023;10:25-8.
115 Sanjay RE, Josmi J, Sasidharanpillai S, Shahin S, Michael CJ, Sabeena S, et al. Molecular epidemiology of enteroviruses associated with hand, foot, and mouth disease in South India from 2015 to 2017. Arch virol. 2022;167:2229-38.
116 Rajaseker C, Sharmila PF, Munisamy M, Kandhasamy V, Sundaramurthy R, Dhodapkar R. Clinico Virological Characterization of Hand, Foot and Mouth Disease in a Tertiary Care Hospital, South India. J glob infect dis. 2023;15:13-8.
117 George GM, Darius-J Daniel H, Mathew L, Peter D, George L, Pulimood S, et al. Changing epidemiology of human enteroviruses (HEV) in a hand, foot and mouth disease outbreak in Vellore, south India. Indian j med microbi. 2022;40:394-8.
118 Gopalkrishna V, Ganorkar N. Epidemiological and molecular characteristics of circulating CVA16, CVA6 strains and genotype distribution in hand, foot and mouth disease cases in 2017 to 2018 from Western India. J med virol. 2021;93:3572-80.
119 Sekaran S, Ganapathy D. Tomato flu outbreak in India: a need for precautionary measures in dental treatment. Int J Surg. 2023;109:511-2.
120 Rai A, Uwishema O, Nicholas A, Abbass M, Uweis L, Arab S, et al. Tomato flu outbreak in India: Why is it an impending public health emergency? Int J Surg. 2023;109:2153-6.
121 Mohanty MC, Dharmapalan D, Vengurlekar P, Varose S, Shete A, Joshi Y, et al. Enteroviruses Associated with Hand Foot and Mouth Disease in Navi Mumbai, Maharashtra, India in 2022. Indian J Pediatr. 2025;92:292-4.
122 Mohta A, Pareek S, Sharma MK, Aggrwal A, Vyas K, Pandey H, et al. Hand Foot Mouth Disease During the SARS-CoV-2 Pandemic: A Multicentric Study. Indian pediatr. 2023;60:394-6.

123 Pattassery SA, Kutteyil SS, Lavania M, Vilasagaram S, Chavan NA, Shinde PA, et al. Molecular epidemiology of hand, foot, and mouth disease in Karnataka, India in 2022. Indian J Med Microbiol. 2023;46:100429.
124 Khajuria A, Saini D, Gupta RK, Sharma A, Babber S. Epidemiological and Clinical Profile of Hand, Foot, and Mouth Disease in Children in a Tertiary Care Center in Jammu. Cureus. 2024;16:e58704.
125 Mamidi P, Mishra B, Panda S, Ray A, Jena D, Biswas V, et al. Identification of a novel Indian clade of Coxsackievirus A16 in Hand, Foot and Mouth disease outbreak in 2022. Future virol. 2024;-:1-12.
126 Tikute S, Deshmukh P, Chavan N, Shete A, Shinde P, Yadav P, et al. Emergence of Recombinant Subclade D3/Y in Coxsackievirus A6 Strains in Hand-Foot-and-Mouth Disease (HFMD) Outbreak in India, 2022. Microorganisms. 2024;12:-.
127 Sriwjitalai W, Wiwanitkit V. Hand foot and mouth disease, tomato flu and new emerging outbreak. J stomatol oral maxi. 2023;124:101274.
128 Suryawanshi H, Sahu M, Singh P. Hand, foot and mouth disease (HFMD): A case report. J Oral Maxillofac Pathol. 2024;28:464-6.
129 Iyer S, Srihari S, Shenoy M. Onycholysis due to Hand, Foot, and Mouth Disease. Archives of Medicine and Health Sciences. 2024;-:-.
130 Girsang RT, Rusmil K, Fadlyana E, Setiabudiawan B, Adrizain R, Mulyadi RP, et al. A serosurvey study of hand, foot and mouth disease in healthy children aged 6 to 71 months old in West Bandung and Bandung Region, Indonesia. BMC Infect Dis. 2025;25:124.
131 Juknis Surveilans Sentinel Revisi 2025. 2025. Available: https://www.researchgate.net/publication/389888857_Juknis_Surveilans_Sentinel_Revisi_2025. Accessed.
132 CDC U. National Enterovirus Surveillance System (NESS). 2025. Available: https://www.cdc.gov/ness/about/index.html. Accessed.
133 NESS Surveillance Data _ National Enterovirus Surveillance System _ CDC. 2025. Available: https://www.cdc.gov/ness/data-vis/. Accessed.
134 Zhu P, Ji W, Li D, Li Z, Chen Y, Dai B, et al. Current status of hand-foot-and-mouth disease. J biomed sci. 2023;30:15.
135 Pearson D, Basu R, Wu XM, Ebisu K. Temperature and hand, foot and mouth disease in California: An exploratory analysis of emergency department visits by season, 2005-2013. Environ res. 2020;185:109461.
136 Messacar K, Spence-Davizon E, Osborne C, Press C, Schreiner TL, Martin J, et al. Clinical characteristics of enterovirus A71 neurological disease during an outbreak in children in Colorado, USA, in 2018: an observational cohort study. Lancet Infect Dis. 2020;20:230-9.
137 Russell NG, Kessler R. A Hand-Foot-and-Mouth Disease Outbreak in an Atypical Population of College Students. J Prim Care Community Health. 2024;15:21501319241266506.
138 Spotts PH. Outbreak of Hand, Foot, and Mouth Disease Among University Residential Students. J am board fam med. 2024;37:513.

139 Machado RS, de Sousa IP, das Chagas Júnior WD, Ferreira JL, Lopes DP, Justino MCA, et al. Molecular evolution of coxsackievirus A6 associated with atypical hand, foot, and mouth disease in Northern Brazil in 2019. Arch Virol. 2025;170:99.
140 Justino MCA, da S Mesquita D, Souza MF, Farias FP, Dos S Alves JC, Ferreira JL, et al. Atypical hand-foot-mouth disease in Belém, Amazon region, northern Brazil, with detection of coxsackievirus A6. J clin virol. 2020;126:104307.
141 Luchs A, Azevedo LS, Souza EV, Medeiros RS, Souza Y, Teixeira DLF, et al. Coxsackievirus A6 strains causing an outbreak of hand-foot-and-mouth disease in Northeastern Brazil in 2018. Rev inst med trop sp. 2022;64:e16.
142 Carmona RCC, Machado BC, Reis FC, Jorge AMV, Cilli A, Dias AMN, et al. Hand, foot, and mouth disease outbreak by Coxsackievirus A6 during COVID-19 pandemic in 2021, São Paulo, Brazil. J clin virol. 2022;154:105245.
143 Xavier JPO, Junior J. Onychomadesis secondary to hand-foot-and-mouth disease: report of two cases. An bras dermatol. 2020;95:266-8.
144 Tomba Ngangas S, Bisseux M, Jugie G, Lambert C, Cohen R, Werner A, et al. Coxsackievirus A6 Recombinant Subclades D3/A and D3/H Were Predominant in Hand-Foot-And-Mouth Disease Outbreaks in the Paediatric Population, France, 2010-2018. Viruses. 2022;14:1078.
145 Cohen R, Béchet S, Gelbert N, Frandji B, Vie Le Sage F, Thiebault G, et al. New Approach to the Surveillance of Pediatric Infectious Diseases From Ambulatory Pediatricians in the Digital Era. Pediatr infect dis j. 2021;40:674-80.
146 Mirand A, Cohen R, Bisseux M, Tomba S, Sellem FC, Gelbert N, et al. A large-scale outbreak of hand, foot and mouth disease, France, as at 28 September 2021. Eurosurveillance. 2021;26:2100978.
147 Mirand A, Henquell C, Archimbaud C, Ughetto S, Antona D, Bailly JL, et al. Outbreak of hand, foot and mouth disease/herpangina associated with coxsackievirus A6 and A10 infections in 2010, France: a large citywide, prospective observational study. Clin microbiol infec. 2012;18:E110-8.
148 Luciani L, Morand A, Zandotti C, Piorkowski G, Boutin A, Mazenq J, et al. Circulation of enterovirus A71 during 2019-2020, Marseille, France. J med virol. 2021;93:5163-6.
149 Bendig JW, Fleming DM. Epidemiological, virological, and clinical features of an epidemic of hand, foot, and mouth disease in England and Wales. Commun Dis Rep CDR Rev. 1996;6:R81-6.
150 Kamau E, Lambert B, Allen DJ, Celma C, Beard S, Harvala H, et al. Enterovirus A71 and coxsackievirus A6 circulation in England, UK, 2006-2017: A mathematical modelling study using cross-sectional seroprevalence data. PLoS Pathog. 2024;20:e1012703.
151 Kamau E, Nguyen D, Celma C, Blomqvist S, Horby P, Simmonds P, et al. Seroprevalence and Virologic Surveillance of Enterovirus 71 and Coxsackievirus A6, United Kingdom, 2006-2017. Emerg infect dis. 2021;27:2261-8.
152 Sinclair C, Gaunt E, Simmonds P, Broomfield D, Nwafor N, Wellington L, et al. Atypical hand, foot, and mouth disease associated with coxsackievirus A6

infection, Edinburgh, United Kingdom, January to February 2014. Eurosurveillance. 2014;19:pii=20745.

153 Joyce AM, Hill JD, Tsoleridis T, Astbury S, Berry L, Howson-Wells HC, et al. Coxsackievirus A6 U.K. Genetic and Clinical Epidemiology Pre- and Post-SARS-CoV-2 Emergence. Pathogens. 2024;13:-.

154 Bednarska NG, Smith S, Bardsley M, Loveridge P, Byford R, Elson WH, et al. Trends in general practitioner consultations for hand foot and mouth disease in England between 2017 and 2022. Epidemiol Infect. 2025;153:e22.

155 Tang JW, Barer MR, Iqbal A, Hamal S, Mohamedanif T, Tipping LF, et al. Kerala Tomato Flu - A Manifestation of Hand Foot and Mouth Disease. Pediatr infect dis j. 2022;41:e501-e3.

156 Bujaki E, Farkas Á, Rigó Z, Takács M. Distribution of enterovirus genotypes detected in clinical samples in Hungary, 2010-2018. Acta microbiol imm h. 2020;67:201-8.

157 Deézsi-Magyar N, Zsidei G, Kiss N, Novák B, Mezősi-Csaplár M, Tarcsai KR, et al. Evaluating enterovirus diversity among symptomatic patients in Hungary during and after easing the COVID-19 lockdown. Virol J. 2025;22:204.

158 Cabrerizo M, Tarragó D, Muñoz-Almagro C, Del Amo E, Domínguez-Gil M, Eiros JM, et al. Molecular epidemiology of enterovirus 71, coxsackievirus A16 and A6 associated with hand, foot and mouth disease in Spain. Clin microbiol infec. 2014;20:O150-6.

159 Martínez-López N, Muñoz-Almagro C, Launes C, Navascués A, Imaz-Pérez M, Reina J, et al. Surveillance for Enteroviruses Associated with Hand, Foot, and Mouth Disease, and Other Mucocutaneous Symptoms in Spain, 2006-2020. Viruses. 2021;13:-.

160 Andrés C, Guasch E, Piñana M, Fernandes P, Gimferrer L, Esso DV, et al. Recombinant CV-A6 strains related to hand-foot-mouth disease and herpangina at primary care centers (Barcelona, Spain). Future microbiol. 2019;14:499-507.

161 Taravilla CN, Pérez-Sebastián I, Salido AG, Serrano CV, Extremera VC, Rodríguez AD, et al. Enterovirus A71 Infection and Neurologic Disease, Madrid, Spain, 2016. Emerg infect dis. 2019;25:25-32.

162 Del Giudice P. Enterovirus A71 Infection and Neurologic Disease, Madrid, Spain, 2016. Emerg infect dis. 2020;26:1638.

163 Santangelo OE, Gianfredi V, Provenzano S, Cedrone F. Digital epidemiology and infodemiology of hand-foot-mouth disease (HFMD) in Italy. Disease trend assessment via Google and Wikipedia. Acta Biomed. 2023;94:e2023107.

164 Scorrano G, Russo M, Prezioso G, Sensi SL, Chiarelli F. A case of pediatric Guillain-Barré syndrome following hand-foot-and-mouth disease and the need for Brighton criteria revision in children. Acta neurol belg. 2023;123:2397-9.

165 Di Lella E, Angelini F, Campagnano S, Messineo D, Drudi FM. An unusual location of hand, foot and mouth disease. J ultrasound. 2022;25:361-4.

166 Lizasoain A, Mir D, Martínez N, Colina R. Coxsackievirus A10 causing hand-foot-and-mouth disease in Uruguay. Int j infect dis. 2020;94:1-3.

167 Lizasoain AM, Natalia; de Mora, Carla; Rodríguez, Edivia; Ledezma, Nathalie; Colina, Rodney;. Identification of the Emerging C1-like Lineage of Enterovirus A71 in Two Uruguayan Children with Hand-Foot-and-Mouth Disease and Neurological Complications. Viruses. 2024;16.
168 Ndiaye N, Teixeira DD, Dia N, Da Silva Leite CC, Fall G, Furtado UD, et al. An outbreak of atypical hand, foot and mouth disease associated Coxsackievirus A6 in children from Cape Verde, 2023. Virol J. 2025;22:48.
169 Itani TM, Chalapa VI, Slautin VN, Imangaliev BS, Kungurtseva MS, Patrusheva AK, et al. Circulation of non-polio enteroviruses in the Ural Federal District and Western Siberia in 2023: the return of an old foe? Arch Virol. 2025;170:110.
170 Giljača S, Maris S, Rančić N, Mrvaljević M, Mrvaljević Z. Epidemiological characteristics of outbreaks of hand, foot and mouth disease in kindergartens in Belgrade during the period from 2015 to 2019. Zdravstvena zaštita. 2021;50:1-12.
171 Prćić S, Matić A, Matić M, Radulović A, Gajinov Z. Hand, Foot, and Mouth Disease in Children: Clinical Characteristics of an Outbreak in Novi Sad, Serbia. Acta dermatovener cr. 2023;31:24-8.
172 Machain-Williams C, Dzul-Rosado AR, Yeh-Gorocica AB, Rodriguez-Ruz KG, Noh-Pech H, Talavera-Aguilar L, et al. Detection of hand, foot and mouth disease in the yucatan peninsula of Mexico. Infect Dis Rep. 2014;6:5627.
173 Javadi M, Nejati A, Yousefi M, Mahmoodi M, Shoja Z, Shahmahmoodi S. First seroepidemiological investigation of human enterovirus 71 in Iran. Iran j microbiol. 2021;13:502-8.
174 Griffin L, Rafferty S, Ahmad K. Hand, foot and mouth disease: is it time for an update? Clin exp dermatol. 2023;48:1277-9.
175 Dumaidi K, Al-Jawabreh A, Zraiqi A, Fashafsha A, Dumaidi A. First Report of Hand, Foot, and Mouth Disease (HFMD) Outbreak in the West Bank, Palestine: Molecular Characterization of Coxsackievirus A16 (CV-A16). Can j infect dis med. 2025;2025:9133821.
176 Arredondo-Nontol R, Arredondo-Nontol M, Castillo-Peña L, Vertiz EA, Gómez GL, Reto N. Onychomadesis secondary to mouth, hand, and foot disease: Case report. Biomedica. 2025;45:190-6.
177 Alakrash L, Barakeh M, AlQahtani WI, AlKanaan RK. Recurrent Hand, Foot, and Mouth Disease in a Saudi Girl. Cureus. 2024;16:e51813.
178 Sapia EY, Maroni C, Groisman C, Kromer H, Lihue Rojo G, Dastugue M, et al. [Atypical hand-foot-mouth disease virus genotyping in a pediatric hospital in Buenos Aires city, Argentina]. Arch argent pediatr. 2020;118:e199-e203.
179 Hoffmann AJ, Latrous M, Lam JM. Atypical hand-foot-and-mouth disease. Can med assoc j. 2020;192:E69.
180 Ooi MH, Wong SC, Lewthwaite P, Cardosa MJ, Solomon T. Clinical features, diagnosis, and management of enterovirus 71. Lancet Neurol. 2010;9:1097-105.
181 Jagadish A, Paladugula A, Notta S, Notta N, Shah R. A Case of Hand-Foot-and-Mouth Disease in an Adult Male. Cureus. 2023;15:e42670.

182 Martins JA, Morais C, Ferreira P, Baptista A. Hand, Foot, and Mouth Disease in a Patient With Psoriasis: A Case Report. Cureus. 2025;17:e80348.
183 Kano Y, Shigehara Y. Hand-foot-and-mouth disease in an adolescent. Am j med sci. 2024;367:e63-e4.
184 Park S, Inaba Y, Tsuruta K, Sugiura K. Hand-foot-and-mouth disease in an elderly adult caused by coxsackievirus A4. J dermatol. 2024;51:e239-e40.
185 Pham KNO, Duong MC, Vo DN, Ho DTN. Hand, foot and mouth disease with encephalomyelitis in adult: A case report. Diagn Microbiol Infect Dis. 2025;112:116832.
186 Sulasmia, Djawad K, Lauren G. Hand, Foot and Mouth Disease in Immunocompetent Adult with Severe Oral Manifestation. Serbian Journal of Dermatology and Venerology. 2020;12:87-91.
187 Manley DP. An adult presentation of a childhood disease. Jaapa. 2021;34:31-3.
188 Peters JF. 75-year-old man • recent history of hand-foot-mouth disease • discolored fingernails and toenails lifting from the proximal end • Dx? J fam practice. 2023;72:138-9.
189 Azari S, Wolfman D, Smith A. Hand, foot, and mouth disease presenting with a testicular mass in an adult. Can j urol. 2024;31:11854-7.
190 Kamler J, Piehl M, Chenevert L, Truong J. Adult presentation of hand, foot, and mouth disease. Vis J Emerg Med. 2022;29:101437.
191 Jahangiri M, Padarti A, Kilgo WA. Post-infectious Transverse Myelitis Secondary to Hand, Foot, and Mouth Disease in a Pregnant Daycare Worker. Cureus. 2024;16:e56159.
192 Milad D, Antaki F, Hammamji K. Unilateral acute idiopathic maculopathy in hand, foot, and mouth disease. Can J Ophthalmol. 2024;59:e176.
193 Ciccarese G, Broccolo F, Fidanzi C, Serviddio G, Drago F. Atypical hand foot and mouth disease related Coxsackievirus-A10 infection in an adult patient. Travel med infect di. 2024;59:102716.
194 Ciccarese G, Broccolo F, Serviddio G, Drago F. Clinical, diagnostic features and complications of hand, foot, and mouth disease caused by coxsackievirus A6 in children and adults. J med virol. 2023;95:e28405.
195 Marletta DA, Beretta AE, Genovese G, Marzano AV. Bullous hand, foot, and mouth disease. Ital j dermatol vene. 2022;157:284-5.
196 Faatz H, Lommatzsch C, Wilming P, Lommatzsch A. [Hand, foot and mouth disease-associated maculopathy: a 2-year course]. Ophthalmologe. 2020;117:798-801.
197 Ikink R, Houwing RH. [Hand-foot-mouth disease in an adult: a clinical presentation caused by an atypical viral agent]. Ned Tijdschr Geneeskd. 2022;166:-.
198 Roshanth N, Christensen LF, Chakari KJ, Mansour I. Acute unilateral maculopathy after hand, foot and mouth disease. Ugeskr Laeger. 2023;185:V12220788.
199 Radunsky K, Boltz A, Vécsei-Marlovits VP. Ocular manifestation of hand, foot, and mouth disease : A case series. Wien med wochenschr. 2024;174:337-41.

200 Di Prinzio A, Bastard DP, Torre AC, Mazzuoccolo LD. Hand, foot, and mouth disease in adults caused by Coxsackievirus B1-B6. An bras dermatol. 2022;97:321-5.
201 Farah M, El Chaer F, El Khoury J, El Zakhem A. Erythema multiforme-like hand, foot, and mouth disease in an immunocompetent adult: a case report. Int j dermatol. 2020;59:487-9.
202 Flipo R, Isnard C, Coutard A, Martres P, Dumas M, Blum L, et al. [Atypical hand, foot and mouth disease in adults: A note on 6 cases]. Ann dermatol vener. 2020;147:857-61.
203 Kritikos A. Atypical hand, foot and mouth disease with eczematiform presentation in an adult patient. Int j infect dis. 2021;105:397-8.
204 Afonso C, Almeida A. Hand, Foot, and Mouth Disease in Adults. Cureus. 2023;15:e48387.
205 Teixeira A, Torres Lima D, Almeida Pereira A, Amaral-Silva M, Miguéns AC. Guillain-Barré Syndrome Following Hand, Foot, and Mouth Disease in an Adult Patient. Cureus. 2023;15:e45423.
206 Barrett SC, Bhat NN, Bindiganavile SH, Lee AG. Postinfectious Optic Neuritis After Hand-Foot-Mouth Disease. J neuro-ophthalmol. 2021;41:e351-e3.
207 Chen Z, Jiang C, Cheng X, Ma L, Xin Y, Liu T, et al. Secondary hypogonadism following hand, foot, and mouth disease in an adult: a case report and review of literature. BMC Infect Dis. 2022;22:56.
208 Liu C, Xu M, Li SW, Sun XG. [A case of hand, foot and mouth disease in adults who first diagnosed with conjunctivitis]. Zhonghua Yan Ke Za Zhi. 2021;57:139-41.
209 Falk ZE, Malik KJ. Unilateral Acute Idiopathic Maculopathy in A Patient with Hand foot mouth disease: A Case Report. Retin Cases Brief Rep. 2022;16:540-2.
210 Yen CY, Fang IM. Unilateral acute idiopathic maculopathy related to hand-foot-mouth disease: Case report and literature review. Taiwan j ophthalmol. 2024;14:133-6.
211 Wang WY, Chiu SH. Coxsackievirus A6-Induced Atypical Hand-Foot-Mouth Disease. Jama dermatol. 2024;160:769-70.
212 Alam MR, Rokaya N, Mahat S, Upadhyaya A, Rokaya P. A Rare Presentation of Hand, Foot, and Mouth Disease During Pregnancy. Cureus. 2022;14:e28401.
213 Giachè S, Borchi B, Zammarchi L, Colao MG, Ciccone N, Sterrantino G, et al. Hand, foot, and mouth disease in pregnancy: 7 years Tuscan experience and literature review. J matern-fetal neo m. 2021;34:1494-500.
214 Nakasone R, Ogi M, Kawamura A, Miyake O, Kido T, Abe S, et al. Vertical Transmission of Coxsackievirus A6 with Severe Congenital Pneumonia/Sepsis. Int J Environ Res Public Health. 2023;20:2843.
215 Hussain K, Chawla S, Muir D, Roberts N. Clinical manifestations of coxsackievirus A6: what a dermatologist needs to know. Int j dermatol. 2023;62:e113-e5.
216 Kim YJ, Kim TG. Pseudomembranous conjunctivitis with hand, foot and mouth disease in a pregnant woman : a case report. BMC Ophthalmol. 2021;21:113.

217 Kabele P, Mojhová M, Smíšková D. Hand-foot-mouth disease in puerperium. Ces gynekol. 2022;87:47-9.
